# A model of dopamine and serotonin-kynurenine metabolism in cortisolemia: Implications for depression

**DOI:** 10.1371/journal.pcbi.1008956

**Published:** 2021-05-10

**Authors:** Felipe Dalvi-Garcia, Luis L. Fonseca, Ana Tereza R. Vasconcelos, Cecilia Hedin-Pereira, Eberhard O. Voit

**Affiliations:** 1 Bioinformatics Lab, National Laboratory for Scientific Computing, Petrópolis, Rio de Janeiro, Brazil; 2 School of Medicine and Surgery, Federal University of the State of Rio de Janeiro, Rio de Janeiro, Rio de Janeiro, Brazil; 3 Department of Biomedical Engineering, Georgia Institute of Technology, Atlanta, Georgia, United States of America; 4 Center of Health Sciences, Federal University of Rio de Janeiro, Rio de Janeiro, Rio de Janeiro, Brazil; 5 Oswaldo Cruz Institute, Oswaldo Cruz Foundation, Rio de Janeiro, Rio de Janeiro, Brazil; US Army Medical Research and Materiel Command: US Army Medical Research and Development Command, UNITED STATES

## Abstract

A major factor contributing to the etiology of depression is a neurochemical imbalance of the dopaminergic and serotonergic systems, which is caused by persistently high levels of circulating stress hormones. Here, a computational model is proposed to investigate the interplay between dopaminergic and serotonergic-kynurenine metabolism under cortisolemia and its consequences for the onset of depression. The model was formulated as a set of nonlinear ordinary differential equations represented with power-law functions. Parameter values were obtained from experimental data reported in the literature, biological databases, and other general information, and subsequently fine-tuned through optimization. Model simulations predict that changes in the kynurenine pathway, caused by elevated levels of cortisol, can increase the risk of neurotoxicity and lead to increased levels of 3,4-dihydroxyphenylaceltahyde (DOPAL) and 5-hydroxyindoleacetaldehyde (5-HIAL). These aldehydes contribute to alpha-synuclein aggregation and may cause mitochondrial fragmentation. Further model analysis demonstrated that the inhibition of both serotonin transport and kynurenine-3-monooxygenase decreased the levels of DOPAL and 5-HIAL and the neurotoxic risk often associated with depression. The mathematical model was also able to predict a novel role of the dopamine and serotonin metabolites DOPAL and 5-HIAL in the ethiology of depression, which is facilitated through increased cortisol levels. Finally, the model analysis suggests treatment with a combination of inhibitors of serotonin transport and kynurenine-3-monooxygenase as a potentially effective pharmacological strategy to revert the slow-down in monoamine neurotransmission that is often triggered by inflammation.

## Author summary

According to the World Health Organization, major depressive disorder (MDD) was in 2014 the fourth leading cause of disability in people between the ages of 15 and 44 years. MDD is responsible for about 1 million suicides per year and associated with a number of other medical conditions such as coronary disease, diabetes, and Alzheimer’s disease. While MDD has been studied for a long time, molecular details of its pathophysiology are still scarce. Computational models offer a powerful opportunity to assist neuropsychiatric disorder research, as they permit the representation of large sets of physiological, cellular and biochemical phenomena through mathematical equations that can be simulated in a very efficient fashion and have the capacity to provide testable hypotheses. Here, we introduce a computational model of relevant biochemical pathways associated with high levels of circulating stress hormone, as they are observed in MDD. The model captures known observations well and demonstrates how increased levels of internally produced toxic agents, such as various kynurenines, DOPAL and 5-HIAL, can lead to dysregulation of key enzymes. These insights suggest new hypotheses for model-driven experiments, as well as novel potential targets for pharmacological intervention.

## Introduction

Affective disorders alter the mood of an individual. According to the Diagnostic and Statistical Manual of Mental Disorders-V, these disorders are typically classified as depressive or bipolar [1] if they are pathological or intense and persistent. Most common among them is major depressive disorder (MDD). Its symptoms include depressive mood, anhedonia, reduced energy, feelings of guilt and/or low self-esteem, sleep problems, changes in appetite, irritability, lack of concentration and bouts of anxiety [2].

According to the World Health Organization, MDD is responsible for about 1 million suicides per year and expected to be the second leading cause of disability in 2020 and the first in 2040 [2,3]. Research during the past decade has focused on links between depression and the development of other medical conditions, such as coronary heart disease [4], diabetes [5] and Alzheimer’s disease [6]. Up to 64% of recovered patients may suffer recurrent episodes of MDD [7], and only about 30 to 35% of adults treated with antidepressants go into remission [8]. Despite these disturbing statistics and the considerable impact of MDD on health and society, the biological basis for the pathophysiology of MDD is still obscure [9].

Different biochemical theories have suggested that imbalances in the levels of biogenic amines, such as dopamine (DA) and serotonin (5-HT), are involved in the etiology of psychiatric disorders like schizophrenia, attention-deficit/hyperactivity disorder, and depression [10–14]. These imbalances in dopaminergic and serotonergic systems are, in turn, likely to affect the chemical balance within the entire neurotransmitter system [15,16] and, as a consequence, are presumably not the only causes for depression. Instead, factors beyond changes in the metabolism of these monoamines likely contribute to the pathogenesis of MDD as well. As a pertinent example, many studies have shown that the influence of persistent, high levels of circulating stress hormones can be a potent trigger of MDD [17,18]. Cortisol (CORT) in humans, or corticosterone in rodents, is one of the hormones that have been directly associated with decreases in dopamine and serotonin levels. The subsequent imbalances in neurotransmission can have different outcomes, depending on the affected brain regions [19].

Impairments in neurotransmitter metabolism due to changes in CORT have been reported to play a key role in the prefrontal cortex (PFC) and can lead to the development of MDD [20–22]. One reason is neuroanatomical: dopaminergic and serotonergic cell bodies are located in other regions, but their nerve terminals are projected towards the PFC where they interact with each other. These terminals possess the entire necessary cellular machinery for synthesis, release, reuptake and degradation of neurotransmitters [23]. As a result, the overlap in dopaminergic and serotonergic axon terminals in the PFC and their metabolic autonomy are important features that suggest a focused investigation of these terminals toward a deeper understanding of the neurochemical processes that are involved in the onset of MDD.

Another contributor to MDD appears to be the kynurenine (KYN) pathway (KP), which constitutes a side branch of tryptophan metabolism through which tryptophan can be channeled away from the serotonin pathway. Although KYN was discovered at the end of the 19^th^ century, long before serotonin, KYN and its metabolites have received increased attention only during the past decade, particularly due to their links to inflammation, the immune system and a variety of neurological conditions [24]. Moreover, it has been reported that KYN derivatives can impair the metabolism of aldehydes that result from the catabolism of dopamine and serotonin, and furthermore regulate glutamate neurotransmission by affecting the N-methyl-D-aspartate (NMDA) receptor [25,26].

Over the years, animal models have been used to study the neuropathophysiology of psychiatric disorders, such as MDD [14]. In particular, the association between stress and the etiology of depression has been investigated with experiments that induce depressive behavior by increasing the level of CORT in an animal through a stress protocol [27,28]. Unfortunately, experiments in animal models are expensive, both in terms of direct costs and also in terms of experimental effort. Additionally, considerations of ethics have become very demanding regarding the use and restrictions of animal models [29]. These factors suggest a search for valid alternative or parallel approaches, notwithstanding the undeniable fact the traditional paradigm of investigation has greatly advanced science and clinical understanding and will be a mainstay throughout the foreseeable future.

One potential alternative is computational modeling, whose power has increased enormously in recent years, both due to striking advances in computing and the availability of very rich molecular datasets. The core of any computational approach is the representation of a biomedical system through mathematical equations, often in the format of ordinary differential equations (ODEs). Once such a mathematical model is assembled, parameterized and coded in algorithmic software, simulations and other explorations of numerous scenarios are cheap and fast. Although these types of “experiments” must always be followed up with biological or clinical validation studies, they often “weed out” erroneous hypotheses, help us explain and predict an organism’s functioning, and guide the design of targeted experiments that advance the field and may eventually lead to novel pharmaceutical interventions.

Employing such a modeling approach, our overall goal in the present study is to deepen our understanding of the interactions within neurotransmitter systems that contribute to the etiology of clinical depression. This goal is pursued through computational simulations with a new mathematical model that elucidates the key components driving the dynamic interactions among the dopaminergic and serotonergic pathways on the one hand and kynurenine metabolism and the role of CORT on the other. The computational analyses focus on chronic stress scenarios and are specifically designed to explain the consequences of changes in these interactions for the onset of MDD.

## Results

### Conceptual model and dynamic model equations

**Fig 1:**
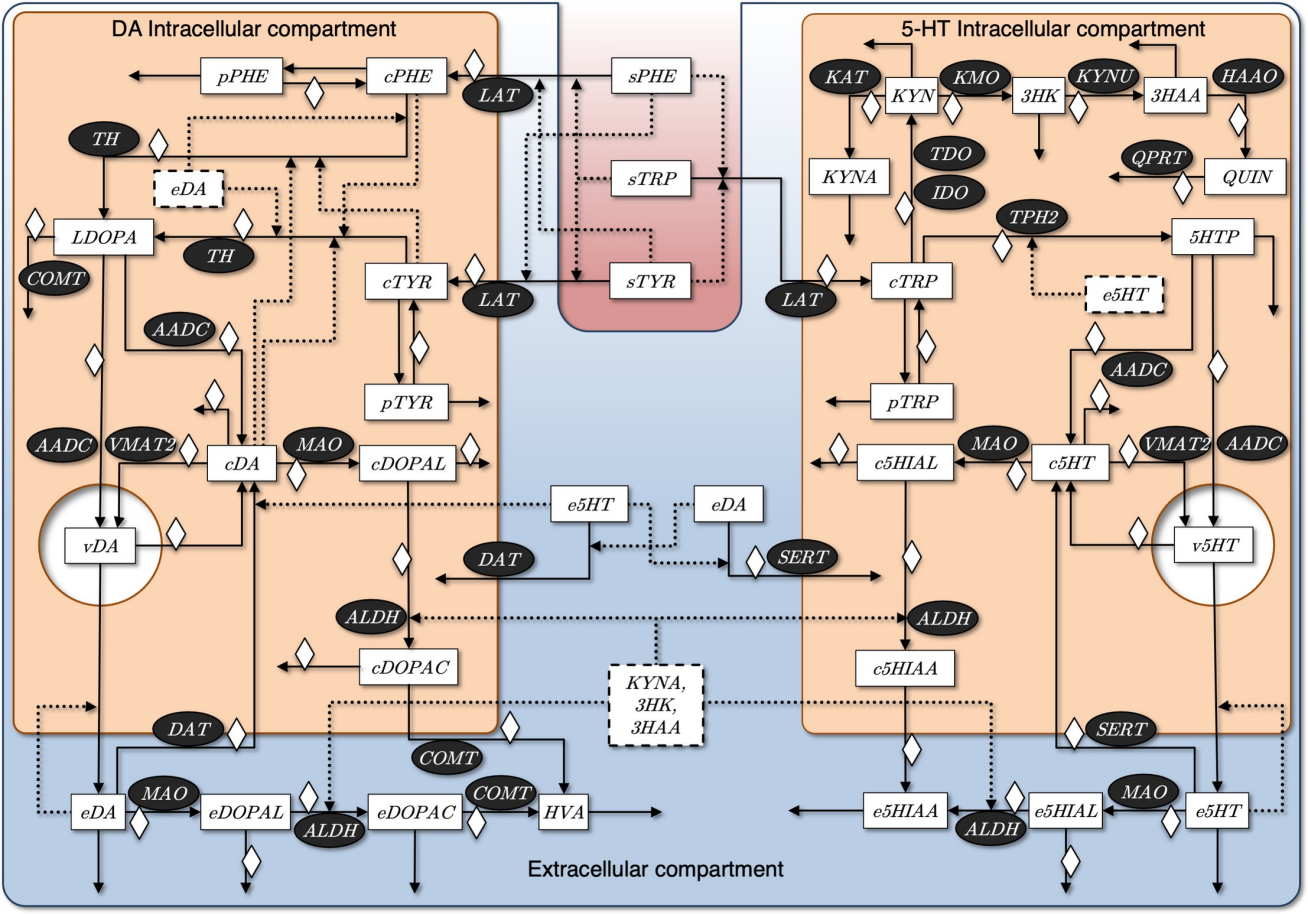
Conceptual, simplified model of pathways associated with MDD and their interactions in the presynaptic DA and 5-HT terminals. The model accounts for separate cytosolic and vesicular compartments in the two terminals, which share the same extracellular space. The latter is an important “collective” location for processes that take place outside the neurons, in particular, in the synaptic cleft or in glial cells. Metabolites are represented with white boxes and participating enzymes with black ellipses. Metabolites in dashed boxes are important but not necessarily located in the compartment where they are represented. Prefixes *s*, *c*, *v*, *e*, and *p* refer to metabolites in the serum, cytosol, vesicles, extracellular space and pool of proteins, respectively. Cortisol/corticosterone (CORT) is represented by a white diamond. Serum tyrosine, phenylalanine, tryptophan, enzymes and CORT are independent variables that remain constant during a given experiment. The strongest inhibitory effects are represented with dotted lines. Abbreviations: 3-HAA, 3-hydroxyanthranilic acid; 3-HK, 3-hydroxykynurenine; 5-HIAA, 5-hydroxyindoleacetic acid; 5-HIAL, 5-hydroxyindoleacetaldehyde; 5-HT, 5-hydroxytryptamine or serotonin; 5-HTP, 5-hydroxytryptophan; AADC, l-amino acid decarboxylase; ALDH, aldehyde dehydrogenase; COMT, catechol O-methyltransferase; DA, dopamine; DAT, dopamine transporter; DOPAC, 3,4-dihydroxyphenylacetic acid; DOPAL, 3,4-dihydroxyphenylacetaldehyde; HAAO, 3-hydroxyanthranilate 3,4-dioxygenase; HVA, homovanillic acid; IDO, indoleamine 2,3-dioxygenase; KAT, kynurenine aminotransferase; KMO, kynurenine 3-monooxygenase; KP, kynurenine pathway; KYN, kynurenine; KYNA, kynurenic acid; KYNU, kynureninase; LAT, L-type amino acid transporter; L-DOPA, l-3,4-hydroxyphenylalanine; MAO, monoamine oxidase; PHE, phenylalanine; QPRT, quinolinate phosphoribosyltransferase; QUIN, quinolinic acid; SERT, serotonin transporter; TDO, tryptophan-2,3-dioxygenase; TH, tyrosine hydroxylase; TPH2, tryptophan hydroxylase 2; TRP, tryptophan; TYR, tyrosine; VMAT2, vesicular monoamine transporter 2.

The model is conceptually based on the pathway system in Fig 1. The translation of this diagram into a so-called Generalized Mass Action (GMA) model is technically straightforward [30,31] and yields the set of equations shown in S1 Supplement (see also Section Methods). The relative steady-state values for each dependent variable and the values for the independent variables are shown in the Tables A and B in S3 Supplement, respectively.

### Sensitivity analysis

Sensitivity analysis quantifies how numerical changes in model settings affect the dependent variables, which here are metabolite concentrations. Specifically, we analyze a special set of sensitivities, called log gains, that quantify relative changes in the steady-state values of metabolites in response to 10% changes in any of the independent variables. We performed this comprehensive gain analysis for two reasons. First, moderate gains, with plus or minus signs, are generally associated with a valid model, because under normal, healthy conditions, most dependent variables are not expected to vary enormously in response to a small change in any of the independent variables. Thus, the magnitudes of such responses are of interest in systems analysis, and small magnitudes typically indicate model robustness, whereas large magnitudes are often a cause for concern. Expressed differently, the magnitudes provide a qualitative measure of the reliability of the model. Indeed, all gains in our model are reasonable, as it can be seen in S4 Supplement, which demonstrates the mathematical consistence of the model.

### Data-based model validation

Simulation experiments were performed to compare the model output with experimental data and clinical findings reported in the literature. Specifically, we considered perturbations in the key enzymes and transporters, namely monoamine oxidase (MAO), catechol O-methyltransferase (COMT), dopamine transporter (DAT), serotonine transporter (SERT), and tryptophan hydroxylase 2 (TPH2). We also investigated their inhibition, as well as the doubling of the activity of the vesicular monoamine transporter 2 (VMAT2), which is a driver of neurotransmitter dynamics [30,32,33]. For these simulations, the model was set to the control state, *i*.*e*., the kinetic orders for the variable CORT were set to zero to represent baseline levels of glucocorticoids (absence of chronic stress). The main results are summarized in Table 1.

**Table 1:** Metabolite changes predicted by the model in comparison to corresponding values found in the literature. Abbreviations: 5-HT, serotonin; COMT, catechol O-methyltransferase; DA, dopamine; DAT, dopamine transporter; DOPAC, 3,4-dihydroxyphenylacetic acid; HVA, homovanillic acid; MAO, monoamine oxidase; SERT, serotonin transporter; TPH2, tryptophan hydroxylase 2; VMAT2, vesicular monoamine transporter 2.

Experiment	Metabolite	Model	Literature
MAO-A/B^-/-^	Extracellular DA	↑34.4%	*↑*3.2–11.6% [34]
	Total DA	*↑*133%	*↑*12 *−* 200% [35,36]
	Extracellular DOPAC	*↓*88%	*↓*21–78.1% [34]
	Total DOPAC	↓70.7%	↓17 *−* 62% [36]
	HVA	↓64.1%	↓30 *−* 48% [36]
	Total 5-HT	↑34.8%	↑209–700% [35,37]
	Total 5-HIAA	*↓*97%	*↓*81.8% [38]
COMT^-/-^	Extracellular DA	*↑*10.6%	*↑*8.6–52% [39,40]
	Total DA	*↑*39.1%	*↑*40% [41]
	Extracellular DOPAC	*↑*646%	*↑*233–628% [39,40]
	Total DOPAC	*↑*318%	*↑*387% [39]
	HVA	*↓*67%	*↓*100% [39]
DAT^-/-^	Extracellular DA	*↑*119%	*↑*393% [42]
	Total DA	*↓*71.5%	*↓*96% [42]
	Total 5-HT	*↑*3.7%	*↑*3.1% [37]
SERT^-/-^	Extracellular 5-HT	*↑*369%	*↑*450–900% [43–45]
	Total 5-HT	*↓*27.4%	*↓*53.8–76.7% [44,45]
	Total 5-HIAA	*↓*74.8%	*↓*44.4–55.6% [44,45]
TPH2^+/-^	Extracellular 5-HT	*↓*15%	*↓*66.2% [46]
	Total 5-HT	*↓*33.7%	*↓*78.5% [46]
	Total 5-HIAA	*↓*54.3%	*↓*72.2% [46]
Two-fold VMAT2	Extracellular DA	*↑*26.3%	*↑*44% [32]
	Total DA	*↑*102%	*↑*21% [32]

The results in Table 1 demonstrate that the model with its parameterization aligns quite well with biological and clinical observations. In particular, all predicted model responses, without exception, correctly point in the observed direction of findings reported in the literature. In many cases, the magnitudes are captured semi-quantitatively. For larger deviations, the magnitudes are not always captured accurately, which is to be expected from a model that is naturally based on approximations and simplifications. Overall, hallmark perturbations such as SERT inhibition, which is widely used to increase extracellular 5-HT, validate our model, at least in a semi-quantitative manner.

### Model experiments with elevated CORT

**Fig 2:**
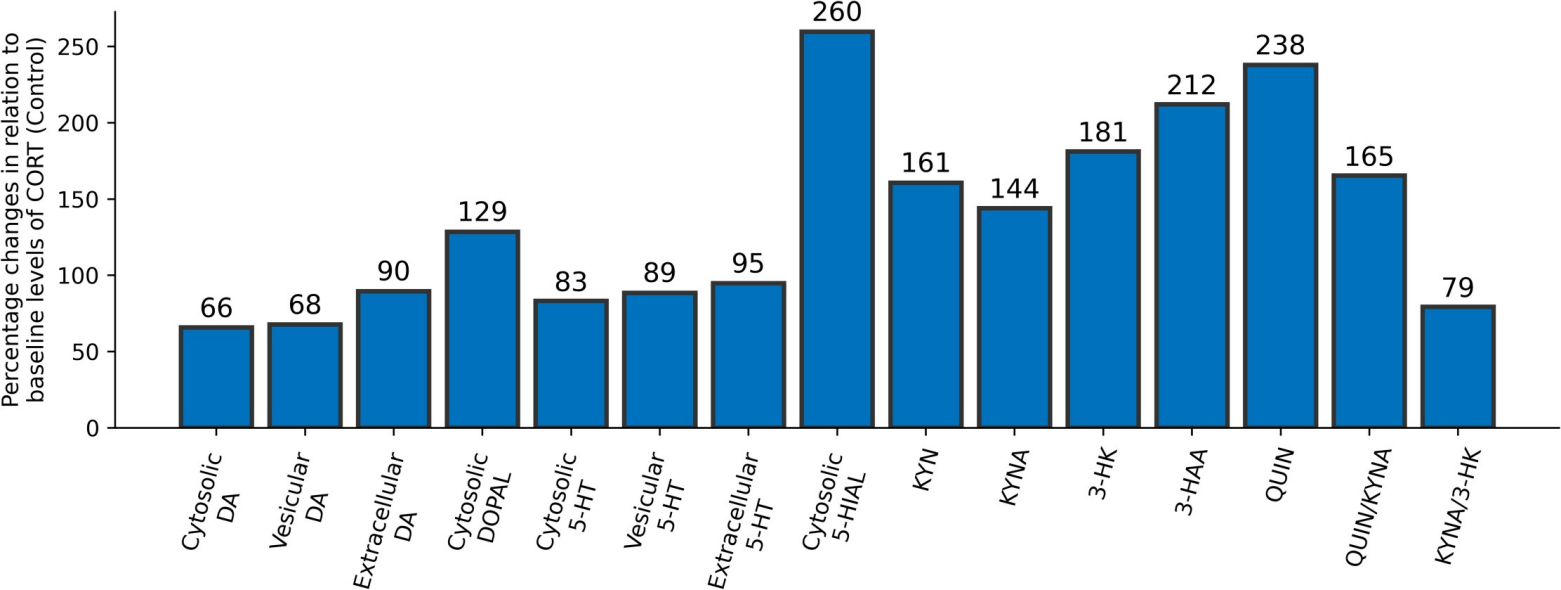
New steady-state values of dependent variables in response to a 50% increase in CORT. The percent changes were calculated in comparison to the corresponding values under control conditions (CORT at baseline of 100%). Also shown are the ratios QUIN/KYNA and KYNA/3-HK, which reflect the balance between levels of key metabolites.

To simulate chronic stress in our experiments, we increased the level of CORT to 150% of its baseline value and computed the new steady-state metabolite concentrations in comparison to their steady-state levels when CORT is operating under physiological conditions (baseline of 100%). Fig 2 displays a profile of the most interesting model components for the case of chronic stress. We also included in this analysis the neurotoxic ratios defined by the relationship between quinilinic acid (QUIN) and kynurenic acid (KYNA), and between KYNA and 3-hydroxykynurenine (3-HK).

The results in Fig 2 are very interesting. First, Badawy & Morgan [26] observed in liver that KYNA, 3-HK and 3-hydroxyanthranilic acid (3-HAA) inhibit the activity of aldehyde dehydrogenase (ALDH), and one might reasonably expect the same to be true in the brain; the corresponding model results are displayed in Table 2. According to Fig 2, CORT stress increases the levels of KYNA (↑44%), 3-HK (↑81%) and 3-HAA (↑112%), which in the model leads to inhibition of the activity of ALDH. This model prediction is supported by the elevated levels of the cytosolic aldehydes 5-hydroxyindoleacetaldehyde (5-HIAL) (↑160%) and 3,4-dihydroxyphenylacetaldehyde (DOPAL) (↑29%), which are substrates of ALDH. Second, the model with settings for stress conditions predicts significant increases in KYN (↑61%) and in the neurotoxic ratio QUIN/KYNA (↑65%), as well as a decrease in the ratio KYNA/3-HK (↓21%).

**Table 2:** Summary of general assumptions made during model design.

Assumption	Comments
The blood-brain barrier (BBB) has just one layer.	The BBB is mainly composed of endothelial cells, which are connected to each other through tight junctions. The only way to cross the BBB is through specific transporters located in the luminal and abluminal membranes (double layer) of the brain capillary endothelia [86,87]. For simplicity, a single layer and one generic type of transporter were considered.
The BBB is the only way for molecules to reach the brain cells.	Since the surface of BBB is more than 1,000 times that of the choroid plexus [88], the BBB area is taken as the exclusive means for a given molecule to access the brain [89].
The L-type amino acid transporter (LAT) is the only one in along the BBB.	Large neutral amino acids (LNAAs), such as tyrosine (TYR), phenylalanine (PHE) and tryptophan (TRP) compete for the same type of transporter, called the L-type amino acid transporter (LAT) [90]. Although different subfamilies of LAT transporters are known, LAT-1 is here assumed to be the sole bidirectional, sodium-independent, high-affinity obligatory exchanger that works for facilitated diffusion.
Only TYR, PHE and TRP cross LAT-1.	In reality, LAT-1 is used not only for passage of TYR, PHE and TRP, but also for transporting brain- chained amino acids (BCAA) [91]. This fact appears to be of secondary importance, and the inhibitory competition of these BCAA in the uptake of TYR, PHE and TRP was not taken into account.
The transport of LNAA occurs directly from the blood serum to the neuronal cytosolic space.	In actuality, LNAAs reach the brain neurons by entering an astrocyte or moving through the extracellular space to a neuron. In the model, these steps are simplified to a single transport step across the BBB through LAT-1, directly to the neuron, as was proposed in the literature [48,49].
Only TYR and PHE affect the dopaminergic pathway, while TRP acts only in the serotonergic pathway.	Uptake of TYR, PHE and TRP can lead into both dopaminergic and serotonergic neurons through intersection points in the two pathways [92–94]. For simplicity, we assume that only TYR and PHE enter dopaminergic neurons, while TRP only enters serotonergic neurons.
The intracellular volume of a nerve terminal is *V*_*i*_ = 1.13x10^-10^ *μl*	Volume estimates are necessary for scaling the results. Direct information is unavailable and the estimates are quite coarse, but appear to be sufficient, as the relative sizes to each other are more important. If such a terminal is taken as a single synaptosome and approximated by a sphere with radius *R*_*i*_ = 300 *nm*, the corresponding intracellular volume is *V*_*i*_ = 1.13x10^-10^ *μl* (notice that this is not the cytosolic volume, but the cell volume as a whole).
The intracellular vesicular volume in a nerve terminal is *V*_*v*_ = 6.7x10^-12^ *μl*	Each terminal contains approximately 200–500 vesicles of approximately 40 *nm* in diameter [95–97]. Taking every vesicle as a sphere with radius *R*_*v*_ = 20 *nm* and multiplying its volume by 200 results in a total vesicular volume *V*_*v*_ = 6.7x10^-12^ *μl*.
The cytosolic volume in a nerve terminal is *V*_*c*_ = 1.06x10^-10^ *μl*	Taking *V*_*i*_ and *V*_*v*_ as above described, the cytosolic volume is, consequently, *V*_*c*_ = *V*_*i*_*−V*_*v*_ = 1.06x10^-10^ *μl*.
The extracellular volume surrounding one nerve terminal is *V*_*e*_ = 1.78x10^-11^ *μl*	The extracellular space surrounding the nerve terminal is assumed to be a 15 *nm*-thick layer [98]. With the above settings, the extracellular space in the immediate vicinity of a terminal, if expressed as a sphere, has the volume *V*_*e*_ = 1.78x10^-11^ *μl*. Considering that there are two terminals, the total shared extracellular volume is 2*V*_*e*_.
Conversion factor from grams of tissue to volume of water: *f*_*c*_ = 0.7 *ml/g*	Units of metabolite concentrations and enzyme kinetic parameters must be consistent to calculate the kinetic orders for power-law approximation. Fluxes and affinities are usually given in molar concentration per unit of time (*mol/l/h*) and molar concentration (*mol/l*), respectively, or in fractions of these units. We used a conversion factor *f*_*c*_ = 0.7 *ml/g* to switch between *mol/g* and *mol/l* [99,100], when necessary.
Every component in the model is assumed to be homogeneously spread throughout the same compartment.	The consideration of spatial heterogeneity would increase the complexity of the model manyfold.
The CORT concentration is assumed to vary proportionally and homogeneously regardless of the compartment.	Since CORT if a lipophilic molecule with low weight, it can cross the BBB by diffusion and quickly disperse toward the concentration equilibrium [101]. It has been shown that changes in CORT levels are easily detected in saliva and urine and that these measurements correlate well with free serum CORT concentrations [102,103]. Thus, absolute values and changes for CORT concentrations taken from saliva, urine or serum are assumed to represent alterations inside the brain cells [104].
Kynurenine metabolites are assumed to vary proportionally and homogeneously regardless of the compartment.	Information regarding KYN-associated metabolite concentrations in dopaminergic and serotonergic neurons is scarce. However, it is fair to assume that these levels are in the nanomolar (*nm*) range within tissues and in the extracellular space. The concentrations of the KYN metabolites of interest are considered uniformly distributed over brain cells and compartments.
Transcriptional and post- translational regulation mechanisms are not considered, so that protein expression is assumed to be directly proportional to gene expression.	Experiments with homozygote and heterozygote mutants in animals do not necessarily result in a 100% and a 50% reduction in protein expression or activity, respectively. However, for simplicity, transcriptional and post- translational mechanisms are ignored, and it is assumed that there is a linear correlation between gene and protein expression [105,106].
Proteins are represented as a fraction of the total amount of protein content in the control situation.	All variables of a mathematical model that in a simulation do not change over time can be explicitly represented as independent variables and defined as constants. Here, this is the case for the proteins involved in enzymatic reactions.
Enzyme concentrations vary to the same degree regardless of the compartment in which they are active.	Although the enzyme concentrations may vary according to the compartment where they are located, it is assumed that their levels change proportionally everywhere, according to the protocol of the experiment, in which proteins are independent variables with values set before each simulation.
Chronic stress is positively correlated to inflammation.	A large body of research has demonstrated an association between social stressors and inflammation. These studies provide evidence of the correlation among stress, depression and the immune system at the levels of proteins, signaling processes and the genome [107].
Chronic stress is positively correlated to cortisolemia.	Chronic stress leads to a reduction in the negative feedback affecting the HPA axis, thereby elevating the levels of CORT over time. In addition, immune cells become less sensitive to the anti-inflammatory effects of CORT, which leads to the so-called “glucocorticoid insensitivity” [108].
Aspects of comorbidities accompanying depression were not taken into account.	MDD usually manifests with other disorders, such as anxiety and post-traumatic stress disorder [109], whereas our model is based on findings concerning depressed patients and animal models of depression.
Neuronal firing was not taken into account.	Although neuronal firing activity plays a key role in neurotransmission and, consequently, in the levels of metabolites, our model focuses only on the biochemical reactions that regulate the monoamine systems.

**Fig 3:**
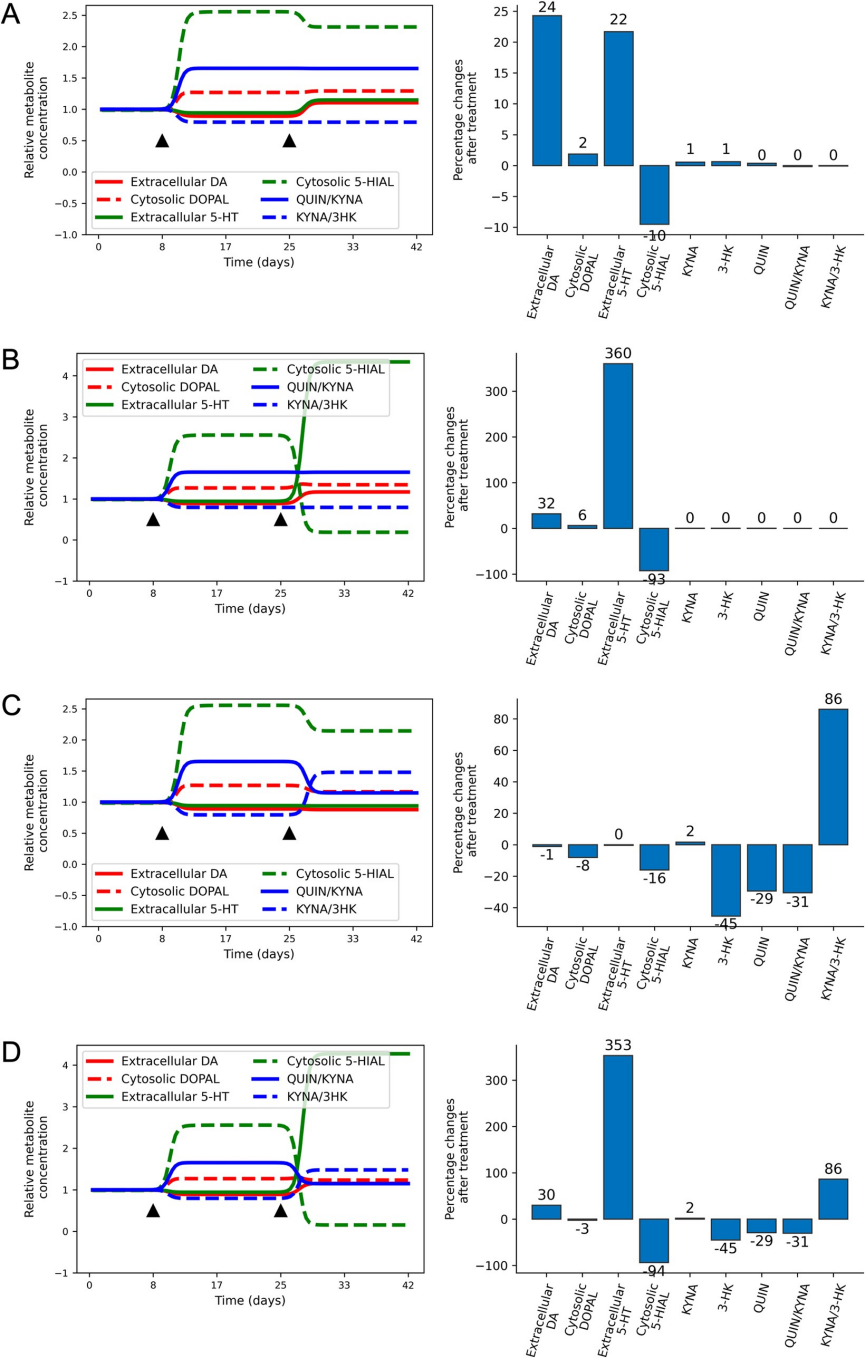
Notable changes in key dependent variables in response to cortisolemia and treatment. (A) Two-fold increase in the activity of VMAT2. (B) 95% Inhibition of the transporter SERT. (C) 50% Inhibition of the activity of enzyme KMO. (D) Combined inhibition of SERT (↓95%) and KMO (↓50%). Time courses are shown on the left and percent changes after treatment on the right of each panel. CORT is increased by 50% at day 8 and “treatment” starts at day 25 (black triangles).

Finally, the model allows us to test interventions that could become potential therapeutic strategies, such as: increase VMAT2 activity (Fig 3A); inhibit SERT (Fig 3B); inhibit kynurenine 3-monooxygenase (KMO) (Fig 3C); and combine inhibition of SERT and KMO (Fig 3D). In addition, Table 2 summarizes the effects of the various simulated “treatments” on key metabolite levels under high levels of CORT. The differences between treated and untreated cases in Table 2 are used in the following discussion.

A two-fold increase in the activity of VMAT2 elevates the levels of monoamines (vesicular, extracellular and total), which is desirable. However, the level of the cytosolic 5-HIAL remains too high (Fig 3A), with a difference of only ↓10% in comparison to the untreated situation. Also, no significant changes in the levels of kynurenine metabolites were observed, especially in 3-HK and QUIN, which are neurotoxic.

By contrast, the inhibition of SERT leads to a strong increase in extracellular 5-HT (↑360%) and a decrease in the level of the cytosolic aldehyde 5-HIAL (↓93%), but not the levels of cytosolic DOPAL, which is actually slightly increased (↑6%) in comparison to the untreated system (Fig 3B). No relevant changes were observed in the kynurenine metabolites.

Since 3-HK is also neurotoxic and serves as an early precursor for the production of QUIN, it is important to find the right balance between the levels of KYNA, 3-HK and QUIN. Along these lines, a simulation with our model demonstrates that inhibition of KMO by 50% reduces the increase in the ratio of QUIN/KYNA (↓31%) and increases KYNA/3-HK (↑86%). Moreover, this strategy causes a small decrease in the level of cytosolic DOPAL (↓8%). Unfortunately, the level of the cytosolic aldehyde 5-HIAL remains high (Fig 3C), even with a decrease of 16%. Notice also that there is no increase in the levels of the extracellular monoamines.

According to the model, the combined inhibition of SERT and KMO not only increases the levels of extracellular 5-HT (↑353%), but also of extracellular DA (↑30%) in comparison to the untreated CORT state (Fig 3D). Although the change in cytosolic DOPAL is relatively low (↓3%), cytosolic 5-HIAL declined 94%. Besides decreasing the levels of aldehydes, this intervention also decreases the neurotoxic ratio, QUIN/KYNA (↓31%), and increases the KYNA/3-HK ratio (↑86%).

## Discussion

We have developed and parameterized a dynamic model to test the impact of elevated cortisol/corticosterone levels and their consequences on serotonin, dopamine and kynurenine pathways, which are known to be associated with the etiology of MDD. Previous models have investigated the interplay of dopaminergic and serotonergic pathways in different contexts, such as the influence of firing and the roles of autoreceptors [47–50]. However, to the best of our knowledge, this is the first mathematical model integrating all pertinent biochemical pathways into a model of depression. Altering parameters in key components of these metabolic pathways generated model predictions that were confirmed by available experimental data; these predictions are therefore acceptable for testing in increased glucocorticoid conditions. High glucocorticoid conditions lead to a detrimental decrease in serotonin production, which is typical of MDD, and were damaging to the activation of the KP. Most importantly, our model predicts significant increases in the cytosolic aldehydes DOPAL and 5-HIAL, in addition to imbalances in the QUIN/KYNA and KYNA/3-HK ratios, which are directly related to neurotoxicity. Therefore, our model suggests the hypothesis that increased levels of DA and 5-HT catabolism, DOPAL and 5HIAL may be important contributors to chronic depression, as they lead to the activation of neurotoxic effects.

According to our model, increased levels of CORT are associated with significant increases in the concentrations of KYNA, 3-HK, and 3-HAA. The increased levels of DOPAL and 5-HIAL reflect evidence of an inhibited ALDH, which in turn is connected to an increased activity of MAO, which becomes evident in the decay of the overall levels of monoamines in our simulation. It is known that these aldehydes are extremely reactive and toxic for the neuron, and rodent studies have demonstrated that DOPAL and 5-HIAL lead to the oligomerization of α-synuclein (αS) [51,52]. Interestingly, recent findings indicate increased levels of αS in the serum of patients with MDD [53], as well as increased amounts of αS-DOPAL oligomers that impair the function of synaptic vesicles, induce DA leakage and further reduce neurotransmission [54].

Studies in humans as well as animal models suggest that αS oligomers lead to mitochondrial dysfunction, and this impairment has been shown to be mediated by the innate immune system and is related to the pathophysiology of MDD [55]. More specifically, fusion or fission processes seem to be especially affected by αS, causing mitochondrial fragmentation [56]. Intriguingly, ALDH is a protein associated with mitochondria, which supports the speculation that its impaired function possibly leads to neurotoxicity that often accompanies cortisolemia. This conclusion is in agreement with a recent study showing that chronic stress does not seem to change the expression of ALDH but can damage the signaling pathway involved with the function of this enzyme in the HPC and PFC [57].

Oxidative stress is the initial step of lipid peroxidation. Here, it yields aldehydes that reduce the level of 3,4-dihydroxyphenylacetic acid (DOPAC) and elevate DOPAL to abnormal levels by inhibiting ALDH activity [58,59]. Evidence of pro-inflammatory cytokines triggering oxidative stress have led to the suggestion of the so-called “oxido-neuroinflammation hypothesis” for the pathogenesis of MDD [60]. The increases in aldehydes and in neurotoxic kynurenine metabolites with high levels of corticosteroids shown by our model support this hypothesis.

It is well known that glutamate can induce neurotoxicity and neuronal death through its agonistic activity on the NMDA receptor. Furthermore, depending on its concentration, KYNA can act on the glycine and/or glutamate modulatory binding sites of the NMDA receptor playing a neuroprotective role by inhibiting the NMDA receptor activity. Conversely, several studies have demonstrated that QUIN stimulates the NMDA receptor, acting as its agonist [25,61], and inhibits its uptake by glial cells, which augments the availability of glutamate [62] and neurotoxicity. Our model does not explicitly include astrocytes and their interactions with the monoaminergic systems, but it could be interesting to explore how glial cells might expand insights gained here.

Elevated levels of these neurotoxic metabolites and of circulating CORT have been correlated with a reduction in HPC volume and dendritic atrophy of its nerve terminals, as observed in MDD patients [63–66]. It has specifically been shown that the increased concentration of 3-HK is associated with neuronal apoptosis in the HPC [67] and that excess extracellular glutamate is related to reduction in dendritic growth [68]. In addition, since HPC is known for having the highest glucocorticoid binding activity of any brain structure [69], the hippocampal control over the hypothalamic-pituitary-adrenal axis is mediated by CORT [70]. These findings are consistent with the widely reported neuropsychological deficits due to hippocampal impairments and volume loss that were observed in untreated MDD patients [71–73].

Our results point to the accumulation of 3-HK and toxic imbalance in the ratio QUIN/KYNA under cortisolemia, besides the increased levels of DOPAL and 5-HIAL. Such dysfunctions are due to changes caused by cortisol in the activity of enzymes in the KP and the monoaminergic system, such as MAO, indoleamine 2,3-dioxygenase (IDO), tryptophan-2,3-dioxygenase (TDO), kynurenine aminotransferase (KAT), and 3-hydroxyanthranilate 3,4-dioxygenase (HAAO). Hence, we propose that all these mechanisms can cause the neuronal death or atrophy in the HPC and PFC as they are observed in depressed patients.

In terms of possible therapeutic targets, our model suggests that overactivating VMAT2 expression twice, a strategy proposed as a therapeutic intervention to re-establish the physiological levels of DA in patients with Parkinson’s diseased or bipolar disorder [32], does not seem to be an efficient approach in comparison to the untreated system, at least with regard to higher levels of glucocorticoids. Also, we observed that the levels of aldehydes and kynurenine metabolites did not change to a meaningful degree, therefore maintaining neurotoxicity.

The model was able to mimic the effect of inhibiting SERT, which leads to a consistent increase in extracellular levels of 5-HT and decreased levels of cytosolic 5-HIAL. By using three classes of pharmacological approaches, two of them interacting with norepinephrine, one of them a SSRI, Martín-Hernández et al. (2019) demonstrated an increase in QUIN/KYNA ratio in the frontal cortex of rats under mild chronic stress and a return to the baseline situation after treatment with antidepressants [74]. However, they found a decrease in the expression of HAAO, although not in all KAT isoforms, which implies that antidepressant treatment can reduce the neurotoxic risk ratio, but at the cost of increasing the levels of KYNA, whose levels are positively correlated to cognitive impairment found in patients with schizophrenia [75]. Moreover, higher levels of 3-HK increase the neuronal vulnerability in patients with Huntington’s disease [76]. Since 3-HK is also neurotoxic and is an early precursor in the production of QUIN, it is essential to find a proper balance between the levels of KYNA and 3-HK and QUIN.

Our current model focuses on DA and 5-HT but by and large ignores the roles of other neurotransmitter systems, such as the role of norepinephrine metabolism in MDD [77]. It could therefore be interesting to expand the current model toward an inclusion of this pathway and explore the so-far ill-characterized molecular mechanisms involved in the interactions between the monoamine system, antidepressants and KP. Such an expansion is currently not feasible, due to many gaps in information, but could be intriguing once experimental studies provide additional data.

Although it seems to be intuitive and straightforward to propose a treatment based on the inhibition of IDO to slow down the KP, it has been shown that such an approach is related to pro-carcinogenic side effects [78]. Also, our gain analysis did not identify a significant impact of IDO on 3-HK, 3-HAA and QUIN. Nonetheless, along these lines, we propose the inhibition of KMO as a possible adjuvant intervention. The corresponding simulation with our model demonstrates that inhibition of KMO is able to reduce QUIN/KYNA while increasing KYNA/3-HK, which is in agreement with other findings in the literature [79]. Interestingly, it has been shown that some KMO inhibitors are more effective in reducing QUIN and 3-HK in situations of strong immune activity [80]. According to our model, the combined strategy of inhibiting SERT and KMO seems to be promising. Hence, we propose the further exploration of KMO inhibitors, in combination with SERT inhibitors, for controlling the production of neurotoxic kynurenine metabolites that is due to the well-known shift in tryptophan metabolism under chronic stress.

In conclusion, our model is the first to suggest that high corticoids trigger an increase in the levels of neurotoxic aldehydes DOPAL and 5-HIAL, which are directly derived from DA and 5-HT catabolism, and that this increase may contribute to chronic depression. This hypothesis implies that the interaction between KP and the dopaminergic and serotonergic catabolic pathways might be an important therapeutic target in MDD. The neurotoxic risk ratio QUIN/KYNA is increased when the level of CORT is elevated, probably leading to glutamate excitoxicity by activation of NMDA receptors. This chain of events may be a key component of PFC neuronal atrophy observed in patients with MDD. To counteract these effects, the computational simulations using classical inhibitors for serotonin and kynurenine pathways suggest that a therapeutic strategy combining SERT and KMO inhibitors would be more effective than SERT inihibition alone. More generally, the recognition of the systemic nature of multiple interacting factors that are involved in MDD and lead to prolonged symptoms and possible brain damage is a fundamental step forward in the development of more efficacious therapeutic approaches.

## Methods

### Pathway structure

The unusual cellular architecture of the PFC includes dopaminergic and serotonergic terminals, even though the cell bodies are located elsewhere. It is therefore reasonable to model the metabolic pathways of dopamine and serotonine-kynurenine in the axon terminals of PFC neurons. The essential details to be included in the model were identified based on literature information, with emphasis on the most relevant metabolic pathways governing DA, 5-HT and KYN within the PFC. Many kinetic features of the reactions were taken from biological databases, such as BRENDA [81], KEGG [82] and MetaCyc [83]. In addition, a few terrific models of some of the pathways of pertinence are available, sometimes in the form of detailed differential equation models. For instance, models are available for the dopamine system [76–77] and the role of levodopa [80], as well as the serotonin pathway [78, 81] and tryptophan and kynurenine metabolism [79]. These models contain much information, some of which we were able to adapt for our purposes. Furthermore, since 5-HT and KYN have tryptophan as a common precursor, KYN metabolism was modeled inside the serotonergic terminal, although most evidence places KYN within the microglia and astrocytes [84,85]. This simplifying assumption seemed reasonable considering that a scientific model is always an abstraction of a complex phenomenon and that we are not concerned about spatial features in this analysis. An overview of the map of reactions and key features within their compartments is shown in Fig 1.

### General and specific assumptions

For purposes of unit conversions, formulation and simulations, some quantitative and qualitative assumptions were made in order to facilitate the understanding of the processes and to render the chemical and physical quantities coherent. General assumptions and their biological rationale are summarized in Table 2.

A few additional assumptions are particular to each pathway, depending on the specifics of the metabolites involved in the reactions and the compartments where these reactions take place. The central assumptions and inferences taken from the literature regarding the dopaminergic and the serotonergic-kynurenine pathways are presented in Table 3. Concentration values for dependent variables at the steady state are listed in Table A in S3 Supplement, while explanations of other values are presented in the following subsections.

**Table 3:** Summary of the specific assumptions used during model design.

Assumption	Comments
TYR, PHE and TRP inhibit each other.	Since these LNAA compete for the transport through the BBB, they reduce their competitors’ net fluxes through LAT-1.
TYR and PHE have just two fates: protein synthesis or synthesis of L-DOPA.	About 90% of the TYR and PHE are used for protein synthesis and 10% for the synthesis of L-DOPA [110]. Furthermore, 90% of the production of LDOPA uses TYR as substrate, since TYR is the preferred substrate of TH.
It is assumed that about 80% of L-DOPA is committed to the synthesis of DA and the remaining 20% is methylated by COMT.	This assumption is reasonable because the majority of L-DOPA is decarboxylated to DA.
At steady state, the concentration of L-DOPA is 10% of the level of cytosolic DA.	Exact proportions are unknown, but similar assumptions were made for other models in the literature [33,48].
Vesicular DA and 5-HT correspond to 98% of the intracellular DA and 5-HT, while the remaining 2% is in the cytosol.	It is generally assumed, and implemented in other models, that most of the intracellular DA is located in the vesicles of dopaminergic neurons [33,48]. The same assumption is made here for serotonergic neurons.
Homovanillic acid (HVA) is considered to exist only in the extracellular space.	The proposed model does not include astrocytes and other glial cells that are involved with the dynamics of HVA. However, COMT is active in the DAergic neurons, so that HVA can also be directly influenced by cytosolic DOPAC.
TRP is assumed to have only three fates: protein synthesis, synthesis of 5- HTP or kynurenine.	It is assumed that about 10% of TRP is used to protein synthesis, whereas 35% is committed to the synthesis of 5-HTP and the remaining 55% is used in the synthesis of kynurenine [79].
It is assumed that about 80% of 5-HTP are committed toward the synthesis of serotonin.	Although 5-HTP plays several peripheral roles, the vast majority is used for the biosynthesis of 5-HT [111].
At the steady state, the concentration of 5-HTP is 10% of the cytosolic serotonin.	A similar assumption is made for DA and L-DOPA.
*eDA/e5HT* = 1	According to the literature, the ratio of *eDA/e5HT* is between 0.71 and 2, with a tendency toward the lower level [112–115].
*iDA/i5HT =* 0.5	According to the literature, 0.5 < *iDA/i5HT* < 0.8, with a tendency toward the lower level [27,116–118].
*i5HT/e5HT =* 850	This value was taken from Adell *et al*. [119].
*e5HIAA/e5HT =* 800	The extracellular concentration of 5-HIAA is much higher than the extracellular concentration of 5-HT [120].
*i5HIAA/e5HIAA =* 0.5	This value was taken from Adell *et al*. [119]. Note that i5HIAA is the tissue concentration of 5-HIAA and had to be converted to cytosolic concentration.
*iDOPAC/eDOPAC =* 0.5	A similar assumption was made for the serotonergic metabolites, intra- and extracellular 5-HIAA. Note, again, that *iDOPAC* is the tissue concentration of DOPAC and had to be converted to cytosolic concentration, since DOPAC is not present in the vesicle compartment.
*cDOPAL/cDOPAC =* 200	DOPAL has been quantified as the precursor aldehyde of DOPAC in the *substantia nigra* of human brains [121]. We adopted the same ratio for the extracellular compartment (*eDOPAL/eDOPAC* = 200).
Cytosolic DA and 5-HT inhibit their own synthesis.	DA inhibits its own synthesis by competing with the cofactor tetrahydrobiopterin (BH4) for the binding site of the enzyme TH [122,123]. We assumed that 5-HT inhibitis its own synthesis by means of a similar competing mechanism.
D_2_ is the only auto-receptor whose activity was taken into account; extracellular DA modulates TH activity through D_2_.	No other receptors that modulate DA synthesis and release were taken into account, since our study focuses on the presynaptic dopaminergic nerve terminal, where D_2_ is most prevalent.
5-HT_1B_ is the only autoreceptor whose activity was taken into account; extracellular 5-HT modulates TPH2 activity through 5-HT_1B_.	No other receptors that modulate 5-HT synthesis and release were taken into account, since our work focuses on the presynaptic serotonergic nerve terminal, where 5-HT_1B_ is most prevalent.
Extracellular DA competes with extracellular 5-HT for SERT	Although all biogenic monoamine transporters are promiscuous, only the competition of DA for SERT was considered, since the affinity of 5-HT for DAT is much lower than DA for SERT [124–126].
TPH2, AADC and VMAT2 have a functional and physical coupling similar to TH, AADC and VMAT2	It has been shown that TH, AADC and VMAT2 have a functional and physicial coupling, so that DA is directly transported into vesicles as soon as it is synthetized [127,128]. We assume that TPH2, AADC and VMAT2 behave in the same way.
KYNA, 3-HK and 3-HAA inhibit the activity of ALDH in the brain.	Even at low concentrations, these kynurenine metabolites can reduce the ALDH activity by 40% or more in the liver [26]. Although ALDH is not the only enzyme involved in the catalysis of DOPAL and 5-HIAL, these aldehydes are mainly oxidized by ALDH [129].
IDO-TDO, MAO, KAT, and HAAO activities are increased under chronic stress.	A substantial body of research supports these assumptions [74,130–132].

One additional assumption made in this work is important and should be discussed in some detail. Namely, some pro-inflammatory cytokines enhance the activity of the enzyme IDO, when high levels of CORT stimulate TDO [133,134], thereby shifting tryptophan from the biosynthesis of 5-HT to the production of kynurenine [135]. Also, it has been shown that the expression of TPH2 is inhibited by methylation in rat brains under stress [136]. Taken together, these observations imply that during an inflammatory response and increased levels of CORT due to chronic stress, not only are the levels of tryptophan available for 5-HT synthesis diminished, but there is also a genetic regulatory mechanism that decreases the expression of the rate- limiting enzyme TPH2. Although cytokines are not necessarily acting simultaneously, since their levels change over time [137], it was assumed for simplicity that, under chronic stress, the levels of CORT and the levels of proinflammatory cytokines are positively correlated [8,107,108]. To implement this assumption, the variable CORT acts directly on the activity of TDO and indirectly represents the influence of cytokines on the activity of IDO and TPH2, thereby shifting tryptophan from 5-HT to KYN synthesis.

### Modeling framework

The choice of the best possible modeling framework is a challenging task, and a lot has been written about it (e.g., [31,138]). Biochemical Systems Theory (BST; [139,140]) was chosen here as the modeling framework, because it is arguably the least biased dynamic approach and requires very few assumptions (*e*.*g*., [31]). It is furthermore rigorously based on Taylor’s approximation theory of numerical analysis [30,141,142]. Other advantages of power-law models in BST over traditional models include the following.

1. Traditional representations, such as Michaelis-Menten functions, suffer from the well-known problem that they are approximations of mass-action kinetics, which in turn assume operation in a well-mixed, homogeneous medium (see [143]), which of course is not the case in living cells. Thus, the foundation of these wide-spread rate laws is not really present *in vivo*. It may be sufficient, but that is unknown and may differ from case to case. The situation is particularly dire in crowded environments as they exist in living cells [144–146].
2. BRENDA [81] and other databases [82,83] contain ample kinetic information, but the true *K*_*M*_ (let alone *V*_*max*_) values for a specific situation like ours (*i*.*e*., cell terminals in a specific brain section, possibly diseased) are very rarely known. The situation is worse for inhibition and other modulator constants. Furthermore, to formulate a traditional model, the type of mechanism for every inhibition or activation signal needs to be known *a priori*, and if it happens to be allosteric, it is unclear how to represent it mechanistically.
3. Power-law functions may at first appear to be unnecessarily complex and require some getting used to. However, they offer tremendous advantages. First, it is an automatic process to convert a pathway diagram into symbolic power-law equations (*e*.*g*., [31]). Namely, every process in every differential equation is represented with a product of power-law terms consisting of a non-negative rate constant and of every variable affecting the process, raised to a real-valued power. Second, in contrast to traditional functions, this representation is mathematically guaranteed (by Taylor’s theory of numerical analysis) to be correct close to the normal operating point of the cell or organism if the parameter values are chosen correctly. Third, power-laws contain fewer (or at most as many) parameters than most traditional functions: There is one kinetic order per variable affecting a given process and one rate constants. Compare this parsimony with the traditional rate law of a bi-substrate-bi-product reaction with inhibition, which can demand a dozen or more parameter values [147]. Fourth, the kinetic orders in power-law models almost always have values within small ranges (Chapter 5 of [30]) which makes it easy to assign default values if true values are unknown. These default values allow the modeler at least to get started. Sensitivity analysis subsequently shows how important the value of this kinetic order is.
4. Careful side-by-side comparisons of large metabolic models, represented either with traditional functions or with power-laws have demonstrated very little difference in results (*e*.*g*., [148–150]), except for crowded media, where power-laws are clearly superior [144–146].

Of course, the power-law formulation is no panacea and has its germane disadvantages. First, a single term does not model saturation, although steady states are represented well by the equations. Second, the model is by its nature an approximation and usually becomes less accurate as simulations deviate far from a chosen operating point, such as the normal steady state.

BST offers several variants [151], among which we decided for the GMA format, because it reflects the stoichiometric relationships within a metabolic pathway system most intuitively. Specifically, all GMA equations have the following form [31,147]:

$$\begin{array}{l} \frac{dX_{i}}{dt}={\dot{X}}_{i}=\sum_{j}\pm \gamma_{ij}\cdot \prod_{k}X_{k}^{f_{ijk}} \end{array}$$ (1)

Here, the change of each dependent variable *i* over time (*dX_i_*/*dt*) is described as a sum or difference of *j* power-law terms, each of which is composed of a positive rate constant *γ_ij_* and of *k* variables that directly affect the particular process. Each equation may have different numbers of terms and each power-law may have different variables, so that both indices, *j* and *k*, typically vary throughout the system of ODEs. Each variable *X_i_* is raised to a power *f_ijk_*, called a kinetic order, which are usually in the range of -1 (miminum inhibitory influence) and 1 (maximum activating influence). If a variable is raised to the power *f_ijk_* = 0 the resulting power-law term is 1, and the variable does not affect the term. Like, dependent variables, which may change over time according to the dynamics of the system and have their own differential equation, independent variables, such as enzyme concentrations, are included in each pertinent term. These variables typically do not change during a given computational experiment or simulation, and they are under the control of the experimenter [33,152,153]. Although the notation in Eq 1 looks restrictive, it is worth noting that the overall model structure is enormously rich and can represent any nonlinearities [151].

The equations of our model can be found in S1 Supplement.

Once the structure of the pathway system is known, it is immediately possible to translate this structure with all details into symbolic power-law equations. This “automatic” formulation is a tremendous advantage over other approaches, and the remaining challenge is the task of determining numerical values of all parameters from data or literature information. An intriguing, multi-decade experience is the fact that BST models are so robust that even relatively substantial variations in kinetic orders, of maybe 30%, usually do not compromise the meaning of a model response, as long as the structure of the biological system is correctly represented and all the relevant connections and metabolites are taken into account [33].

### Parameter identification

While the design of a symbolic BST model is straightforward, the estimation of parameter values is a challenge, as it is for all other model types. Where available, quantitative and qualitative experimental or clinical information was converted into parameter values, with methods that have been demonstrated many times (*e*.*g*., [30,31,154]). Information embedded in other types of models was directly converted with purely mathematical means [148–150]. Nonetheless, as is to be expected, not all pertinent information was available, which required us to use default values (see [30,33]) and computational adaptations so that qualitative results matched observations.

Specifically, the model was fit in two phases: the “control state” and the “CORT state”. The control state was defined as a vector of model variables for the value of CORT at its baseline. In a mammalian model under physiological conditions, some of the variables refer to the basal levels of circulating corticosterone. This corticosterone oscillates during the day, but it is generally assumed that the level does not significantly affect the steady states of neurotransmitter metabolites. For calibrating the model at the control state, the variable CORT was fixed as a constant, and data taken from animal experiments found in the literature were used to adjust the default values of kinetic orders in some reactions to fit additional experimental data (see Table C in S2 Supplement).

It is important to note that the minimal effect of corticosterone on neurotransmitters under normal conditions does not hold for chronic stress situations. Thus, in the second phase of model calibration, the CORT state was analyzed. Specifically, to estimate the parameter values related to corticosterone, all kinetic orders determined for the control state were fixed and only the kinetic orders for CORT (increased by a factor greater than 1, *i*.*e*., > 100%) were adjusted based on data from experiments on chronic stress in animal models (see Table C in S2 Supplement). Finally, the rate constants were calculated for the final set of power-law equations according to the methodology described in [30] and based on the flux partitioning ratios taken from other models in the literature [33,47–50,79,155].

In both situations, an additional constrained optimization was performed to achieve an ensenmble of well-fitting models. Initially, several sets of random kinetic orders were generated using Latin Hypercube Sampling (LHS) of 100 *d*-size [156], where *d* is the number of parameters to be optimized. Every set of the Latin hypercube values was tested and the residual mean squared error (MSE) for each parameter combination was calculated. At the end, 5% of the results with the lowest MSE were selected as initial values for a follow-up local search optimization, using the Nelder-Mead algorithm [157]. Among the optimized sets of parameters, a value was chosen that appeared to be clinically most suitable. The equations of our model can be found in S1 Supplement and the final sets of kinetic orders and rate constants are shown in Tables A and B in S2 Supplement, respectively.

### Model validation

Two approaches were used to assess the reasonableness of the model: one strictly mathematical, the other based on data.

The first approach to validating the parameterized model was a typical sensitivity analysis, which allowed us to judge the adequacy and robustness of the mathematical formulation of the system (for different variants of this type of analysis, see [30]). Specifically, we focused on “logarithmic (log) gains”, each of which quantifies the relative (percent) change in the steady-state concentration of a metabolite (dependent variable) in response to a small-percent change in an independent variable, such as an enzyme activity or hormone level. We performed this analysis for 1% and 10% variations.

The second approach used the comparison of predicted results against data not used to calibrate the model. Specifically, if experimental data are too scarce to allow rigorous parameter estimation and model validation, other strategies must be found to validate the model. In our case, for example, the complexity of the brain is so enormous that there is no guarantee that neuronal metabolism in the *substantia nigra* or the striatum is the same, or even similar, in the PFC [158]. In particular, there are only a few datasets reporting metabolic measurements in the PFC of animals under chronic stress, and other information to verify the behavior of the model variables does not exist. Thus, the best that can be done is a qualitative or semi-quantitative comparison between the results obtained from our model in specific scenarios and the corresponding results from experiments (*in situ*, *ex vivo*, *in vitro* or computational) that are in some sense similar although not exactly equivalent.

### Simulation framework

The parameterized model was implemented in the free platform Python, version 3.8, and run on a MacBook, dual-core processor Intel Core i7, 1.7 GHz, 8 GB RAM, and 64 bits of operating system.

## Supporting information

S1 Supplement Model equations. (DOCX)

S2 Supplement Results of the parameter estimation, including literature sources. (DOCX)

S3 Supplement Physiological values of dependent and independent variables. (DOCX)

S4 Supplement Sensitivity analysis. (DOCX)
