# Supplementary material for: A model of dopamine and serotonin-kynurenine metabolism in cortisolemia: Implications for depression
Source: PLoS Comput Biol. 2021 May 10;17(5):e1008956. doi: 10.1371/journal.pcbi.1008956 (PMC8136856; doi:10.1371/journal.pcbi.1008956)
Supplement: S1 Supplement — (DOCX) [file pcbi.1008956.s001.docx]

**S1 Supplement. Model equations**

This supplement presents the formulation of the ordinary differential equations that define the model. Each equation represents the temporal change (derivative with respect to time; indicated by dot-notation) of one of the dependent variables, which in most cases code for metabolites. By and large, the right-hand sides of these equations consist of sums and differences of fluxes entering or leaving the system. Each of this flux is named as $V_{b}^{a}$, where $a$ is the source pool of metabolites and $b$ is the target pool. In some cases, the target pool is of no interest, and $b$ is omitted.

|  | $\dot{cTYR}= V_{ctyr}^{styr}+ V_{ctyr}^{ptyr}- V_{ldopa}^{ctyr}- V_{ptyr}^{ctyr}$ | (S1.1) |
| --- | --- | --- |
|  | $\dot{cPHE}= V_{cphe}^{sphe}+ V_{cphe}^{pphe}- V_{ldopa}^{cphe}- V_{pphe}^{cphe}$ | (S1.2) |
|  | $\dot{LDOPA}= V_{ldopa}^{ctyr}+ V_{ldopa}^{cphe}- V_{vda}^{ldopa}- V_{cda}^{ldopa}- V^{ldopa}$ | (S1.3) |
|  | $\dot{cDA}= V_{cda}^{ldopa}+ V_{cda}^{eda}+ V_{cda}^{vda}- V_{vda}^{cda}- V_{cdopal}^{cda}-V^{cda}$ | (S1.4) |
|  | $\dot{vDA}= V_{vda}^{ldopa}+V_{vda}^{cda}-V_{cda}^{vda}-V_{eda}^{vda}$ | (S1.5) |
|  | $\dot{eDA}= V_{eda}^{vda}-V_{cda}^{eda}-V_{edopal}^{eda}-V_{5ht}^{eda}-V^{eda}$ | (S1.6) |
|  | $\dot{cDOPAL}= V_{cdopal}^{cda}-V_{cdopac}^{cdopal}-V^{cdopal}$ | (S1.7) |
|  | $\dot{cDOPAC}= V_{cdopac}^{cdopal}- V_{hva}^{cdopac}- V^{cdopac}$ | (S1.8) |
|  | $\dot{eDOPAC}= V_{edopac}^{edopal}-V_{hva}^{edopac}-V^{edopac}$ | (S1.9) |
|  | $\dot{eDOPAL}= V_{edopal}^{eda}-V_{edopac}^{edopal}-V^{edopal}$ | (S1.10) |
|  | $\dot{HVA}= V_{hva}^{cdopac}+V_{hva}^{edopac}-V^{hva}$ | (S1.11) |
|  | $\dot{pTYR}= V_{ptyr}^{ctyr}-V_{ctyr}^{ptyr}-V^{ptyr}$ | (S1.12) |
|  | $\dot{pPHE}= V_{pphe}^{cphe}-V_{cphe}^{pphe}-V^{pphe}$ | (S1.13) |
|  | $\dot{cTRP}= V_{ctrp}^{strp}+V_{ctrp}^{ptrp}-V_{5htp}^{ctrp}-V_{kyn}^{ctrp}-V_{ptrp}^{ctrp}$ | (S1.14) |
|  | $\dot{5HTP}= V_{5htp}^{ctrp}-V_{v5ht}^{5htp}-V_{c5ht}^{5htp}-V^{5htp}$ | (S1.15) |
|  | $\dot{c5HT}= V_{c5ht}^{5htp}+V_{c5ht}^{v5ht}+V_{c5ht}^{e5ht}-V_{v5ht}^{c5ht}-V_{c5hial}^{c5ht}-V^{c5ht}$ | (S1.16) |
|  | $\dot{v5HT}= V_{v5ht}^{5htp}+V_{v5ht}^{c5ht}-V_{c5ht}^{v5ht}-V_{e5ht}^{v5ht}$ | (S1.17) |
|  | $\dot{e5HT}= V_{e5ht}^{v5ht}-V_{c5ht}^{e5ht}-V_{e5hial}^{e5ht}-V_{da}^{e5ht}-V^{e5ht}$ | (S1.18) |
|  | $\dot{c5HIAL}= V_{c5hial}^{c5ht}-V_{c5hiaa}^{c5hial}-V^{c5hial}$ | (S1.19) |
|  | $\dot{c5HIAA}= V_{c5hiaa}^{c5hial}-V_{e5hiaa}^{c5hiaa}$ | (S1.20) |
|  | $\dot{e5HIAL}= V_{e5hial}^{e5ht}-V_{e5hiaa}^{e5hial}-V^{e5hial}$ | (S1.21) |
|  | $\dot{e5HIAA}= V_{e5hiaa}^{e5hial}+V_{e5hiaa}^{c5hiaa}-V^{e5hiaa}$ | (S1.22) |
|  | $\dot{KYN}= V_{kyn}^{ctrp}-V_{kyna}^{kyn}-V_{3hk}^{kyn}-V^{kyn}$ | (S1.23) |
|  | $\dot{KYNA}= V_{kyna}^{kyn}-V^{kyna}$ | (S1.24) |
|  | $\dot{3HK}= V_{3hk}^{kyn}-V_{3haa}^{3hk}-V^{3hk}$ | (S1.25) |
|  | $\dot{3HAA}= V_{3haa}^{3hk}-V_{quin}^{3haa}-V^{3haa}$ | (S1.26) |
|  | $\dot{QUIN}= V_{quin}^{3haa}-V^{quin}$ | (S1.27) |
|  | $\dot{pTRP}= V_{ptrp}^{ctrp}-V_{ctrp}^{ptrp}-V^{ptrp}$ | (S1.28) |

The fluxes in these equations are represented as power-law terms, to which the parameters were assigned. The values of these parameters were inferred in two steps for CORT conditions (see Section Methods).

|  | $V_{ctyr}^{styr}= \gamma_{1}\cdot{sTYR}^{f_{1,1}}\cdot{sPHE}^{f_{1,2}}\cdot{sTRP}^{f_{1,3}}\cdot LAT \cdot CORT^{f_{1,4}}$ | (S1.29) |
| --- | --- | --- |
|  | $V_{ldopa}^{ctyr}= \gamma_{2}\cdot{cTYR}^{f_{2,1}}\cdot{cPHE}^{f_{2,2}}\cdot{LDOPA}^{f_{2,3}}\cdot{cDA}^{f_{2,4}}\cdot{eDA}^{f_{2,5}}\cdot TH \cdot CORT^{f_{2,6}}$ | (S1.30) |
|  | $V_{cphe}^{sphe}= \gamma_{3}\cdot{sTYR}^{f_{3,1}}\cdot{sPHE}^{f_{3,2}}\cdot{sTRP}^{f_{3,3}}\cdot LAT \cdot CORT^{f_{3,4}}$ | (S1.31) |
|  | $V_{ldopa}^{cphe}= \gamma_{4}\cdot{cTYR}^{f_{4,1}}\cdot{cPHE}^{f_{4,2}} \cdot{cDA}^{f_{4,3}}\cdot{eDA}^{f_{4,4}}\cdot TH \cdot CORT^{f_{4,5}}$ | (S1.32) |
|  | $V_{vda}^{ldopa}= \gamma_{5}\cdot LDOPA^{f_{5,1}} \cdot AADC \cdot VMAT2 \cdot{CORT}^{f_{5,2}}$ | (S1.33) |
|  | $V_{cda}^{ldopa}= \gamma_{6}\cdot LDOPA^{f_{6,1}} \cdot{cDA}^{f_{6,2}}\cdot AADC \cdot{CORT}^{f_{6,3}}$ | (S1.34) |
|  | $V^{ldopa}= \gamma_{7}\cdot LDOPA^{f_{7,1}} \cdot COMT \cdot{CORT}^{f_{7,2}}$ | (S1.35) |
|  | $V^{cda}= \gamma_{8}\cdot{cDA}^{f_{8,1}}\cdot{CORT}^{f_{8,2}}$ | (S1.36) |
|  | $V_{vda}^{cda}= \gamma_{9}\cdot{cDA}^{f_{9,1}}\cdot{vDA}^{f_{9,2}}\cdot VMAT2 \cdot{CORT}^{f_{9,3}}$ | (S1.37) |
|  | $V_{cdopal}^{cda}= \gamma_{10}\cdot cDA^{f_{10,1}} \cdot{cDOPAL}^{f_{10,2}}\cdot MAO \cdot{CORT}^{f_{10,3}}$ | (S1.38) |
|  | $V_{cdopac}^{cdopal}= \gamma_{11}\cdot{cDOPAL}^{f_{11,1}}\cdot{cDOPAC}^{f_{11,2}}\cdot{KYNA}^{f_{11,3}}\cdot3{HK}^{f_{11,4}}\cdot3{HAA}^{f_{11,5}}\cdot ALDH \cdot{CORT}^{f_{11,6}}$ | (S1.39) |
|  | $V^{cdopal}= \gamma_{12}\cdot cDOPAL^{f_{12,1}} \cdot{cDA}^{f_{12,2}}\cdot CORT^{f_{12,3}}$ | (S1.40) |
|  | $V_{edopac}^{edopal}= \gamma_{13}\cdot{eDOPAL}^{f_{13,1}}\cdot{eDOPAC}^{f_{13,2}}\cdot{KYNA}^{f_{13,3}}\cdot3{HK}^{f_{13,4}}\cdot3{HAA}^{f_{13,5}}\cdot ALDH \cdot{CORT}^{f_{13,6}}$ | (S1.41) |
|  | $V^{edopal}= \gamma_{14}\cdot eDOPAL^{f_{14,1}}\cdot CORT^{f_{14,2}}$ | (S1.42) |
|  | $V^{cdopac}= \gamma_{15}\cdot cDOPAC^{f_{15,1}} \cdot{cDA}^{f_{15,2}}\cdot CORT^{f_{15,3}}$ | (S1.43) |
|  | $V_{cda}^{vda}= \gamma_{16}\cdot vDA^{f_{16,1}} \cdot CORT^{f_{16,2}}$ | (S1.44) |
|  | $V_{eda}^{vda}= \gamma_{17}\cdot{vDA}^{f_{17,1}}\cdot{eDA}^{f_{17,2}}$ | (S1.45) |
|  | $V^{eda}= \gamma_{18}\cdot eDA^{f_{18,1}}$ | (S1.46) |
|  | $V_{cda}^{eda}= \gamma_{19}\cdot{eDA}^{f_{19,1}}\cdot{eDA}^{f_{19,2}}\cdot e5{HT}^{f_{19,3}}\cdot DAT \cdot{CORT}^{f_{19,4}}$ | (S1.47) |
|  | $V_{5ht}^{eda}= \gamma_{20}\cdot eDA^{f_{20,1}} \cdot e5{HT}^{f_{20,2}}\cdot SERT \cdot{CORT}^{f_{20,3}}$ | (S1.48) |
|  | $V_{edopal}^{eda}= \gamma_{21}\cdot eDA^{f_{21,1}} \cdot{eDOPAL}^{f_{21,2}}\cdot MAO \cdot{CORT}^{f_{21,3}}$ | (S1.49) |
|  | $V_{hva}^{cdopac}= \gamma_{22}\cdot cDOPAC^{f_{22,1}} \cdot HVA^{f_{22,2}}\cdot COMT \cdot CORT^{f_{22,3}}$ | (S1.50) |
|  | $V_{hva}^{edopac}= \gamma_{23}\cdot eDOPAC^{f_{23,1}} \cdot{HVA}^{f_{23,2}}\cdot COMT \cdot{CORT}^{f_{23,3}}$ | (S1.51) |
|  | $V^{hva}= \gamma_{24}\cdot HVA^{f_{24,1}}$ | (S1.52) |
|  | $V_{ptyr}^{ctyr}= \gamma_{25}\cdot{cTYR}^{f_{25,1}}$ | (S1.53) |
|  | $V_{pphe}^{cphe}= \gamma_{26}\cdot{cPHE}^{f_{26,1}}$ | (S1.54) |
|  | $V_{ctyr}^{ptyr}= \gamma_{27}\cdot pTYR^{f_{27,1}} \cdot CORT^{f_{27,2}}$ | (S1.55) |
|  | $V_{cphe}^{pphe}= \gamma_{28}\cdot pPHE^{f_{28,1}} \cdot CORT^{f_{28,2}}$ | (S1.56) |
|  | $V^{ptyr}= \gamma_{29}\cdot pTYR^{f_{29,1}}$ | (S1.57) |
|  | $V^{pphe}= \gamma_{30}\cdot pPHE^{f_{30,1}}$ | (S1.58) |
|  | $V^{edopac}= \gamma_{31}\cdot eDOPAC^{f_{31,1}}$ | (S1.60) |
|  | $V_{ctrp}^{strp}= \gamma_{32}\cdot{sTYR}^{f_{32,1}}\cdot{sPHE}^{f_{32,2}}\cdot{sTRP}^{f_{32,3}}\cdot LAT \cdot{CORT}^{f_{32,4}}$ | (S1.61) |
|  | $V_{5htp}^{ctrp}= \gamma_{33}\cdot{cTRP}^{f_{33,1}}\cdot{c5HT}^{f_{33,2}}\cdot e5{HT}^{f_{33,3}}\cdot{KYN}^{f_{33,4}}\cdot TPH2 \cdot{CORT}^{f_{33,5}}$ | (S1.62) |
|  | $V_{v5ht}^{5htp}= \gamma_{34}\cdot{5HTP}^{f_{34,1}} \cdot v5{HT}^{f_{34,2}}\cdot AADC \cdot VMAT2 \cdot{CORT}^{f_{34,3}}$ | (S1.63) |
|  | $V_{c5ht}^{5htp}= \gamma_{35}\cdot{5HTP}^{f_{35,1}} \cdot c5{HT}^{f_{35,2}}\cdot AADC \cdot{CORT}^{f_{35,3}}$ | (S1.64) |
|  | $V^{5htp}= \gamma_{36}\cdot5{HTP}^{f_{36,1}}$ | (S1.65) |
|  | $V^{c5ht}= \gamma_{37}\cdot{c5HT}^{f_{37,1}} \cdot{CORT}^{f_{37,2}}$ | (S1.66) |
|  | $V_{c5hial}^{c5ht}= \gamma_{38}\cdot c5{HT}^{f_{38,1}}\cdot c5{HIAL}^{f_{38,2}}\cdot MAO \cdot{CORT}^{f_{38,3}}$ | (S1.67) |
|  | $V_{c5hiaa}^{c5hial}= \gamma_{39}\cdot c5{HIAL}^{f_{39,1}}\cdot c5{HIAA}^{f_{39,2}}\cdot{KYNA}^{f_{39,3}}\cdot3{HK}^{f_{39,4}}\cdot3{HAA}^{f_{39,5}}\cdot ALDH \cdot{CORT}^{f_{39,6}}$ | (S1.68) |
|  | $V_{e5hiaa}^{c5hiaa}= \gamma_{40}\cdot c5{HIAA}^{f_{40,1}}\cdot{CORT}^{f_{40,2}}$ | (S1.69) |
|  | $V_{v5ht}^{c5ht}= \gamma_{41}\cdot c5{HT}^{f_{41,1}}\cdot v5{HT}^{f_{41,2}}\cdot VMAT2 \cdot{CORT}^{f_{41,3}}$ | (S1.70) |
|  | $V_{c5ht}^{v5ht}= \gamma_{42}\cdot v{5HT}^{f_{42,1}} \cdot{CORT}^{f_{42,2}}$ | (S1.71) |
|  | $V_{e5ht}^{v5ht}= \gamma_{43}\cdot v5{HT}^{f_{43,1}}\cdot e5{HT}^{f_{43,2}}$ | (S1.72) |
|  | $V_{c5ht}^{e5ht}= \gamma_{44}\cdot e5{HT}^{f_{44,1}}\cdot e5{HT}^{f_{44,2}}\cdot SERT \cdot{CORT}^{f_{44,3}}$ | (S1.73) |
|  | $V_{da}^{e5ht}= \gamma_{45}\cdot e5{HT}^{f_{45,1}}\cdot{eDA}^{f_{45,2}}\cdot DAT \cdot{CORT}^{f_{45,3}}$ | (S1.74) |
|  | $V^{e5ht}= \gamma_{46}\cdot{e5HT}^{f_{46,1}}$ | (S1.75) |
|  | $V_{e5hial}^{e5ht}= \gamma_{47}\cdot{e5HT}^{f_{47,1}}\cdot e5{HIAL}^{f_{47,2}}\cdot MAO \cdot{CORT}^{f_{47,3}}$ | (S1.76) |
|  | $V_{e5hiaa}^{e5hial}= \gamma_{48}\cdot e5{HIAL}^{f_{48,1}}\cdot e5{HIAA}^{f_{48,2}}\cdot{KYNA}^{f_{48,3}}\cdot3{HK}^{f_{48,4}}\cdot3{HAA}^{f_{48,5}}\cdot ALDH \cdot{CORT}^{f_{48,6}}$ | (S1.77) |
|  | $V^{c5hial}= \gamma_{49}\cdot{c5HIAL}^{f_{49,1}} \cdot c5{HT}^{f_{49,2}}\cdot{CORT}^{f_{49,3}}$ | (S1.78) |
|  | $V^{e5hial}= \gamma_{50}\cdot{e5HIAL}^{f_{50,1}} \cdot{CORT}^{f_{50,2}}$ | (S1.79) |
|  | $V^{e5hiaa}= \gamma_{51}\cdot{e5HIAA}^{f_{51,1}}$ | (S1.80) |
|  | $V_{kyn}^{ctrp}= \gamma_{52}\cdot{cTRP}^{f_{52,1}}\cdot{KYN}^{f_{52,2}}\cdot IDO \cdot TDO \cdot{CORT}^{f_{52,3}}$ | (S1.81) |
|  | $V_{3hk}^{kyn}= \gamma_{53}\cdot{KYN}^{f_{53,1}}\cdot3{HK}^{f_{53,2}}\cdot KMO \cdot{CORT}^{f_{53,3}}$ | (S1.82) |
|  | $V_{3haa}^{3hk}= \gamma_{54}\cdot3{HK}^{f_{54,1}}\cdot3{HAA}^{f_{54,2}}\cdot KYNU \cdot{CORT}^{f_{54,3}}$ | (S1.83) |
|  | $V_{quin}^{3haa}= \gamma_{55}\cdot3{HAA}^{f_{55,1}}\cdot{QUIN}^{f_{55,2}}\cdot HAAO \cdot{CORT}^{f_{55,3}}$ | (S1.84) |
|  | $V_{kyna}^{kyn}= \gamma_{56}\cdot{KYN}^{f_{56,1}}\cdot{KYNA}^{f_{56,2}}\cdot KAT \cdot{CORT}^{f_{56,3}}$ | (S1.85) |
|  | $V^{kyn}= \gamma_{57}\cdot{KYN}^{f_{57,1}}$ | (S1.86) |
|  | $V^{3hk}= \gamma_{58}\cdot{3HK}^{f_{58,1}}$ | (S1.87) |
|  | $V^{knya}= \gamma_{59}\cdot{KYNA}^{f_{59,1}}$ | (S1.88) |
|  | $V^{3haa}= \gamma_{60}\cdot{3HAA}^{f_{60,1}}$ | (S1.89) |
|  | $V^{quin}= \gamma_{61}\cdot{QUIN}^{f_{61,1}}\cdot QPRT \cdot{CORT}^{f_{61,2}}$ | (S1.90) |
|  | $V_{ptrp}^{ctrp}= \gamma_{62}\cdot{cTRP}^{f_{62,1}}$ | (S1.91) |
|  | $V_{ctrp}^{ptrp}= \gamma_{63}\cdot{pTRP}^{f_{63,1}} \cdot{CORT}^{f_{63,2}}$ | (S1.92) |
|  | $V^{ptrp}= \gamma_{64}\cdot{pTRP}^{f_{64,1}}$ | (S1.93) |

The final sets of kinetic orders *f_i,j_* and rate constants *γ_i_* are shown in Tables A and B in S2 Supplement, respectively.

Abbreviations: *3HAA*, 3-hydroxyanthranilic acid; *3HK*, 3-hydroxyanthranilic acid; *5HTP*, 5-hydroxytryptophan; *AADC*, aromatic l-amino acid decarboxylase; *ALDH*, aldehyde dehydrogenase; *c5HT*, cytosolic serotonin; *c5HIAA*, cytosolic 5-hydroxyindoleacetic acid; *c5HIAL*, cytosolic 5-hydroxyindoleacetaldehyde; *cDA*, cytosolic dopamine; *cDOPAC*, cytosolic 3,4-dihydroxyphenylacetic acid; *cDOPAL*, cytosolic 3,4-dihydroxyphenylacetaldehyde; *COMT*, catechol O-methyltransferase; *CORT*, corticosterone/cortisol; *cPHE*, cytosolic phenylalanine; *cTRP*, cytosolic tryptophan; *cTYR*, cytosolic tyrosine; *DAT*, dopamine transporter; *e5HT*, extracellular serotonin; *e5HIAL*, extracellular 5-hydroxyindoleacetaldehyde; *e5HIAA*, extracellular 5-hydroxyindoleacetic acid; *eDA*, extracellular dopamine; *eDOPAC*, extracellular 3,4-dihydroxyphenylacetic acid; *eDOPAL*, extracellular 3,4-dihydroxyphenylacetaldehyde; *HAAO*, 3-hydroxyanthranilic acid dioxygenase; *HVA*, homovanillic acid; *IDO*, indoleamine-2,3-dioxygenase; *KAT*, kynurenine aminotransferase; *KMO*, kynurenine-3-monooxygenase; *KYN*, kynurenine; *KYNA*, kynurenic acid; *KYNU*, kynureninase; *LAT*, l-type amino acid transporter; *LDOPA*, l-3,4-dihydroxyphenylalanine; *MAO*, monoamine oxidase; *pPHE*, phenylalanine in the pool of proteins; *pTRP*, tryptophan in the pool of proteins; *pTYR*, tyrosine in the pool of proteins; *QPRT*, quinolinate phosphoribosyltransferase; *QUIN*, quinolinic acid; *SERT*, serotonin transporter; *sPHE*, serum phenylalanine; *sTRP*, serum tryptophan; *sTYR*, serum tyrosine; *TDO*, tryptophan-2,3-dioxygenase; *TH*, tyrosine hydroxylase; *TPH2*, tryptophan hydroxylase 2; *v5HT*, vesicular serotonin; *vDA*, vesicular dopamine; *VMAT2*, vesicular monoamine transporter 2.
