# Supplementary material for: A model of dopamine and serotonin-kynurenine metabolism in cortisolemia: Implications for depression
Source: PLoS Comput Biol. 2021 May 10;17(5):e1008956. doi: 10.1371/journal.pcbi.1008956 (PMC8136856; doi:10.1371/journal.pcbi.1008956)
Supplement: S2 Supplement — (DOCX) [file pcbi.1008956.s002.docx]

**S2 Supplement. Results of the parameter estimation, including literature sources**

**Table A. Values of kinetic orders and sources.**

| **Kinetic Order** | **Value** | **Kinetic Order** | **Value** |
| --- | --- | --- | --- |
| $f_{1,1}$ | 0.894 [1,2] | $f_{27,1}$ | 1*^a^* |
| $f_{1,2}$ | -0.443 [1,2] | $f_{27,2}$ | 1*^a^* |
| $f_{1,3}$ | -0.384 [1,2] | $f_{28,1}$ | 1*^a^* |
| $f_{1,4}; f_{3,4}; f_{32,4}$ | 1*^a^* | $f_{28,2}$ | 1*^a^* |
| $f_{2,1}$ | -0.642 [3] | $f_{32,1}$ | -0.106 [1,2] |
| $f_{2,2}$ | -0.331 [3] | $f_{32,2}$ | -0.443 [1,2] |
| $f_{2,4}; f_{4,3}$ | -0.125 [2] | $f_{32,3}$ | 0.616 [1,2] |
| $f_{2,5}; f_{4,4}$ | -0.460 [2] | $f_{33,1}$ | 0.190 [18] |
| $f_{2,6};f_{4,5}$ | 1*^a^* | $f_{33,5}$ | -0.387*^a^* |
| $f_{3,1}$ | -0.106 [1,2] | $f_{34,1}; f_{35,1}$ | 1 [9] |
| $f_{3,2}$ | 0.557 [1,2] | $f_{34,2}$ | -0.854*^a^* |
| $f_{3,3}$ | -0.384 [1,2] | $f_{35,2}$ | -0.321*^a^* |
| $f_{4,1}$ | -0.578 [3] | $f_{36,1}$ | 0.963*^a^* |
| $f_{4,2}$ | 0.149 [3] | $f_{37,1}$ | 1*^a^* |
| $f_{5,1}; f_{6,1}$ | 1 [9] | $f_{37,2}$ | -0.5*^a^* |
| $f_{5,2};f_{9,3};$  $f_{34,3};f_{41,3}$ | -0.100*^a^* | $f_{38,1}$ | 0.999 [9] |
| $f_{6,2}$ | -0.361*^a^* | $f_{38,2}$ | -0.002*^a^* |
| $f_{6,3}; f_{35,3}$ | 0.500*^a^* | $f_{39,1}$ | 0.867 [10] |
| $f_{7,1}$ | 1 [14] | $f_{39,2}$ | -0.414*^a^* |
| $f_{7,2}; f_{22,3}; f_{23,3}$ | -1*^a^* | $f_{40,2}$ | 0.547*^a^* |
| $f_{27}$ | 1*^a^* | $f_{41,1}$ | 0.703 [25] |
| $f_{8,1}$ | 0.435*^a^* | $f_{41,2}$ | -0.650*^a^* |
| $f_{8,2}$ | 0.001*^a^* | $f_{42,2}$ | 0.001*^a^* |
| $f_{9,1}$ | 0.857 [17] | $f_{43,2}$ | -0.176 [4] |
| $f_{9,2}$ | -0.650*^a^* | $f_{44,1}$ | 0.997 [6] |
| $f_{10,1}$ | 1 [11] | $f_{44,2}$ | 0.144 [7] |
| $f_{10,2}$ | -0.944*^a^* | $f_{45,1}$ | 0.752 [8] |
| $f_{10,3}; f_{21,3};$  $f_{38,3}; f_{47,3}$ | 1 [20] | $f_{45,2}$ | -0.007 [5] |
| $f_{11,1}, f_{13,1}$ | 0.874 [10] | $f_{47,1}$ | 1 [9] |
| $f_{11,3};f_{13,3};$  $f_{39,3},;f_{48,3}$ | -0.032 [22] | $f_{48,1}$ | 0.802 [10] |
| $f_{11,4}; f_{13,4};$  $f_{39,4}; f_{48,4}$ | -0.102 [22] | $f_{49,2}$ | 0.500*^a^* |
| $f_{11,5}; f_{13,5};$  $f_{39,5}, f_{48,5}$ | -0.213 [22] | $f_{49,3}$ | 0.001*^a^* |
| $f_{11,6}; f_{13,6};$  $f_{39,6}; f_{48,6}$ | -1*^a^* | $f_{50,2}$ | -0.500*^a^* |
| $f_{12,1}$ | 1*^a^* | $f_{52,1}$ | 0.264 [15] |
| $f_{12,3}$ | 1*^a^* | $f_{52,3}$ | 0.700*^a^* |
| $f_{14,1}$ | 1*^a^* | $f_{53,1}$ | 0.998 [16] |
| $f_{14,2}$ | 1*^a^* | $f_{53,3}$ | 0.800*^a^* |
| $f_{15,1}$ | 1*^a^* | $f_{54,1}$ | 0.999 [19] |
| $f_{15,3}$ | 1*^a^* | $f_{54,3}$ | 1*^a^* |
| $f_{16,2}$ | 1*^a^* | $f_{55,1}$ | 0.995 [21] |
| $f_{19,1}$ | 0.993 [5] | $f_{55,3}$ | 0.400*^a^* |
| $f_{19,3}$ | -0.248 [8] | $f_{56,1}$ | 0.998 [23] |
| $f_{19,4}; f_{45,3}$ | -0.100*^a^* | $f_{56,3}$ | 0.001*^a^* |
| $f_{20,1}$ | 1 [6] | $f_{61,1}$ | 0.984 [24] |
| $f_{20,2}$ | -0.003 [6] | $f_{61,2}$ | -0.500*^a^* |
| $f_{20,3}; f_{44,3}$ | 0.001*^a^* | $f_{62,1}$ | 0.920 [13] |
| $f_{21,1}$ | 1 [11] | $f_{63,1}$ | 1*^a^* |
| $f_{22,1}$ | 1 [12] | $f_{63,2}$ | 1*^a^* |
| $f_{23,1}$ | 1 [12] | $f_{18,1}; f_{24,1};{f_{29,1}; f}_{30,1};f_{31,1};$  $f_{46,1};f_{51,1};f_{55,1};{f_{57,1};f}_{58,1};$  $f_{59,1};{f_{60,1};f}_{64,1}$ | 1*^b^* |
| $f_{25,1}$ | 0.587 [13] | $f_{2,3};f_{11,2};f_{13,2};f_{17,2};f_{21,2};$  $f_{22,2};f_{23,2};f_{33,2};{f_{33,3}; f}_{33,4};f_{39,2};$  $f_{47,2};f_{48,2};f_{52,2};f_{53,2};f_{54,2};$  $f_{55,2};f_{56,2}$ | -0.3*^c^* |
| $f_{26,1}$ | 0.709 [13] | $f_{12,2};f_{15,2};f_{16,1};{f_{19,2};f}_{17,1};f_{40,1};$  $f_{42,1};f_{43,1};f_{49,1};f_{50,1} ;$  $f_{27,2}; f_{28,2}; f_{63,2}$ | 0.5*^c^* |

*^a^*Optimization, *^b^*processes assumed to be linear, *^c^*heuristics/default values

**Table B. Values of rate constants.**

| **Rate constant** | **Value** | **Rate constant** | **Value** | **Rate constant** | **Value** | **Rate constant** | **Value** |
| --- | --- | --- | --- | --- | --- | --- | --- |
| $\gamma_{1}$ | 2,340E-01 | $\gamma_{17}$ | 1,233E+03 | $\gamma_{33}$ | 2,820E+03 | $\gamma_{49}$ | 6,021E+00 |
| $\gamma_{2}$ | 1,504E+04 | $\gamma_{18}$ | 4,516E+02 | $\gamma_{34}$ | 2,191E+02 | $\gamma_{50}$ | 4,755E+02 |
| $\gamma_{3}$ | 3,250E-02 | $\gamma_{19}$ | 1,307E+02 | $\gamma_{35}$ | 1,238E-01 | $\gamma_{51}$ | 2,699E+01 |
| $\gamma_{4}$ | 2,156E+00 | $\gamma_{20}$ | 6,480E+00 | $\gamma_{36}$ | 2,546E+03 | $\gamma_{52}$ | 1,812E-04 |
| $\gamma_{5}$ | 2,586E-02 | $\gamma_{21}$ | 6,065E-02 | $\gamma_{37}$ | 8,295E+03 | $\gamma_{53}$ | 1,405E-03 |
| $\gamma_{6}$ | 1,753E-01 | $\gamma_{22}$ | 2,936E+03 | $\gamma_{38}$ | 4,353E-04 | $\gamma_{54}$ | 2,301E-04 |
| $\gamma_{7}$ | 3,600E+03 | $\gamma_{23}$ | 1,904E+03 | $\gamma_{39}$ | 7,427E+02 | $\gamma_{55}$ | 2,516E-02 |
| $\gamma_{8}$ | 1,362E+03 | $\gamma_{24}$ | 4,396E+03 | $\gamma_{40}$ | 1,656E+01 | $\gamma_{56}$ | 5,725E-01 |
| $\gamma_{9}$ | 8,996E+03 | $\gamma_{25}$ | 7,279E+02 | $\gamma_{41}$ | 1,303E+04 | $\gamma_{57}$ | 1,770E+03 |
| $\gamma_{10}$ | 1,058E+00 | $\gamma_{26}$ | 2,169E+01 | $\gamma_{42}$ | 8,495E+00 | $\gamma_{58}$ | 6,357E+01 |
| $\gamma_{11}$ | 1,826E+02 | $\gamma_{27}$ | 4,235E-05 | $\gamma_{43}$ | 7,889E+02 | $\gamma_{59}$ | 4,165E+02 |
| $\gamma_{12}$ | 1,721E-04 | $\gamma_{28}$ | 5,541E-06 | $\gamma_{44}$ | 7,054E+01 | $\gamma_{60}$ | 6,357E+00 |
| $\gamma_{13}$ | 7,806E+01 | $\gamma_{29}$ | 3,811E-01 | $\gamma_{45}$ | 1,105E+01 | $\gamma_{61}$ | 1,515E+00 |
| $\gamma_{14}$ | 6,099E-04 | $\gamma_{30}$ | 4,987E-02 | $\gamma_{46}$ | 4,454E+02 | $\gamma_{62}$ | 1,166E+00 |
| $\gamma_{15}$ | 6,248E-02 | $\gamma_{31}$ | 5,618E+01 | $\gamma_{47}$ | 8,478E-02 | $\gamma_{63}$ | 3,255E-06 |
| $\gamma_{16}$ | 1,430E-02 | $\gamma_{32}$ | 1,078E-01 | $\gamma_{48}$ | 2,815E+02 | $\gamma_{64}$ | 2,929E-02 |

**Table C. Experimental data used for parameter estimation.**

| **Experiment** | **Metabolite** | **Reference** |
| --- | --- | --- |
| VMAT2^+/-^ | Total DA | *↓*43.1 – 48.8% [26] |
|  | Total DOPAC | *↓*2.7 – 38.5% [26] |
| VMAT2^-/-^ | Total DA | *↓*60 – 88.2% [26] |
|  | Total DOPAC | *↓*36.6 – 52.4% [26] |
|  | HVA | *↓*33.4% [26] |
| CORT *↑*19-55% | Total DA | *↓*3.7 – 49.1% [27,28] |
|  | Total DOPAC | *↓*5.5 – 40.6% [28,29] |
|  | HVA | *↓*1.2 – 6.3% [27,28] |
|  | Total 5-HT | *↓*6.1% [30] |
|  | Total 5-HIAA | *↓*3.7% [30] |
|  | KYN | *↑*45.1 – 268% [31,32] |
|  | KYNA | *↑*46.2% [32] |
|  | 3-HK | *↑*35% [31] |
|  | QUIN | *↑*37% [32] |

Abbreviations: 3-HK, 3-hydroxykynurenine; 5-HT, serotonin; 5-HIAA, hydroxyindoleacetic acid; COMT, catechol O-methyltransferase; DA, dopamine; DAT, dopamine transporter; DOPAC, 3,4-dihydroxyphenylacetic acid; HVA, homovanillic acid; KYN, kynurenine; KYNA, kynurenic acid; MAO, monoamine oxidase; QUIN, quinolinic acid; SERT, serotonin transporter; TPH2, tryptophan hydroxylase 2; VMAT2, vesicular monoamine transporter 2.

**References**

1. Smith QR, Momma S, Aoyagi M, Rapoport SI. Kinetics of neutral amino acid transport across the blood-brain barrier. J Neurochem. 1987;49(5):1651–8.

2. Best JA, Nijhout HF, Reed MC. Homeostatic mechanisms in dopamine synthesis and release: a mathematical model. Theor Biol Med Model. 2009;6(1):21.

3. Ribeiro P, Pigeon D, Kaufman S. The hydroxylation of phenylalanine and tyrosine by tyrosine hydroxylase from cultured pheochromocytoma cells. J Biol Chem. 1991;

4. Best JA, Nijhout HF, Reed M. Serotonin synthesis, release and reuptake in terminals: a mathematical model. Theor Biol Med Model. 2010;7(1):34.

5. Ross SB. Synaptic Concentration of Dopamine in the Mouse Striatum in Relationship to the Kinetic Properties of the Dopamine Receptors and Uptake Mechanism. J Neurochem. 1991;

6. Larsen MB, Sonders MS, Mortensen OV, Larson GA, Zahniser NR, Amara SG. Dopamine transport by the serotonin transporter: A Mechanistically distinct mode of substrate translocation. J Neurosci. 2011;

7. Hagan CE, Mcdevitt RA, Liu Y, Furay AR, Neumaier JF. 5-HT1B autoreceptor regulation of serotonin transporter activity in synaptosomes. Synapse. 2012;

8. Norrholm SD, Horton DB, Dwoskin LP. The promiscuity of the dopamine transporter: Implications for the kinetic analysis of [3H]serotonin uptake in rat hippocampal and striatal synaptosomes. Neuropharmacology. 2007;

9. Jebai F, Hanoun N, Hamon M, Thibault J, Peltre G, Gros F, et al. Expression, purification, and characterization of rat aromatic L-amino acid decarboxylase in Escherichia coli. Protein Expr Purif. 1997;

10. Han IO, Joo C. Purification and characterization of the rat liver mitochondrial aldehyde dehydrogenases. Korean Biochem J. 1991;24(4):353–60.

11. Fowler CJ, Benedetti MS. The Metabolism of Dopamine by Both Forms of Monoamine Oxidase in the Rat Brain and Its Inhibition by Cimoxatone. J Neurochem. 1983;

12. Gulliver PA, Tipton KF. THE PURIFICATION AND PROPERTIES OF PIG BRAIN CATECHOL‐o‐METHYLTRANSFERASE. J Neurochem. 1979;

13. Oja SS. INCORPORATION OF PHENYLALANINE, TYROSINE AND TRYPTOPHAN INTO PROTEIN OF HOMOGENATES FROM DEVELOPING RAT BRAIN: KINETICS OF INCORPORATION AND RECIPROCAL INHIBITION. J Neurochem. 1972;

14. Bonifácio MJ, Palma PN, Almeida L, Soares-Da-Silva P. Catechol-O-methyltransferase and its inhibitors in Parkinson’s disease. CNS Drug Reviews. 2007.

15. Littlejohn TK, Takikawa O, Skylas D, Jamie JF, Walker MJ, Truscott RJW. Expression and purification of recombinant human indoleamine 2,3-dioxygenase. Protein Expr Purif. 2000;

16. SAITO Y, HAYAISHI O, ROTHBERG S. Studies on oxygenases; enzymatic formation of 3-hydroxy-L-kynurenine from L-kynurenine. J Biol Chem. 1957;

17. Volz TJ, Hanson GR, Fleckenstein AE. Kinetic analysis of developmental changes in vesicular monoamine transporter-2 function. Synapse. 2006;

18. McKinney J, Knappskog PM, Haavik J. Different properties of the central and peripheral forms of human tryptophan hydroxylase. J Neurochem. 2005;

19. Dang Y, Dale WE, Brown OR. Effects of oxygen on kynurenine-3-monooxygenase activity. Redox Rep. 2000;

20. Slotkin TA, Seidler FJ, Ritchie JC. Effects of aging and glucocorticoid treatment on monoamine oxidase subtypes in rat cerebral cortex: therapeutic implications. Brain Res Bull. 1998;47(4):345–8.

21. Foster AC, White RJ, Schwarcz R. Synthesis of Quinolinic Acid by 3‐Hydroxyanthranilic Acid Oxygenase in Rat Brain Tissue In Vitro. J Neurochem. 1986;

22. Badawy AA-B, Morgan CJ. Tryptophan metabolites as potent inhibitors of aldehyde dehydrogenase activity and potential alcoholism-aversion therapeutic agents. In: International Congress Series. 2007. p. 344–51.

23. Varasi M, Della Torre A, Heidempergher F, Pevarello P, Speciale C, Guidetti P, et al. Derivatives of kynurenine as inhibitors of rat brain kynurenine aminotransferase. Eur J Med Chem. 1996;

24. Foster AC, Whetsell WO, Bird ED, Schwarcz R. Quinolinic acid phosphoribosyltransferase in human and rat brain: Activity in Huntington’s disease and in quinolinate-lesioned rat striatum. Brain Res. 1985;

25. Peter D, Jimenez J, Liu Y, Kim J, Edwards RH. The chromaffin granule and synaptic vesicle amine transporters differ in substrate recognition and sensitivity to inhibitors. J Biol Chem. 1994;

26. Mooslehner KA, Chan PM, Xu W, Liu L, Smadja C, Humby T, et al. Mice with Very Low Expression of the Vesicular Monoamine Transporter 2 Gene Survive into Adulthood: Potential Mouse Model for Parkinsonism. Mol Cell Biol. 2001;

27. Wolkowitz O, Sutton M, Koulo M, Labarca R, Wilkinson L, Doran A, et al. Chronic corticosterone administration in rats: Behavioral and biochemical evidence of increased central dopaminergic activity. Eur J Pharmacol. 1986;

28. Mizoguchi K, Yuzurihara M, Ishige A, Sasaki H, Chui DH, Tabira T. Chronic stress induces impairment of spatial working memory because of prefrontal dopaminergic dysfunction. J Neurosci. 2000;

29. Mitani H, Shirayama Y, Yamada T, Kawahara R. Plasma levels of homovanillic acid, 5-hydroxyindoleacetic acid and cortisol, and serotonin turnover in depressed patients. Prog Neuro-Psychopharmacology Biol Psychiatry. 2006;

30. Roth KA, Mefford IM, Barchas JD. Epinephrine, norepinephrine, dopamine and serotonin: Differential effects of acute and chronic stress on regional brain amines. Brain Res. 1982;

31. Fuertig R, Azzinnari D, Bergamini G, Cathomas F, Sigrist H, Seifritz E, et al. Mouse chronic social stress increases blood and brain kynurenine pathway activity and fear behaviour: Both effects are reversed by inhibition of indoleamine 2,3-dioxygenase. Brain Behav Immun. 2016;

32. Martín-Hernández D, Tendilla-Beltrán H, Madrigal JLM, García-Bueno B, Leza JC, Caso JR. Chronic mild stress alters kynurenine pathways changing the glutamate neurotransmission in frontal cortex of Rats. Mol Neurobiol. 2019;56(1):490–501.
