# Supplementary material for: A model of dopamine and serotonin-kynurenine metabolism in cortisolemia: Implications for depression
Source: PLoS Comput Biol. 2021 May 10;17(5):e1008956. doi: 10.1371/journal.pcbi.1008956 (PMC8136856; doi:10.1371/journal.pcbi.1008956)
Supplement: S3 Supplement — (DOCX) [file pcbi.1008956.s003.docx]

**S3 Supplement. Physiological values of dependent and independent variables**

**Table A. Dependent variables and their steady-state concentrations at the baseline level of CORT.** The values are given in relative units, but are consistent with real concentrations found in the literature.

| **Variable** | **Metabolite** | **Concentration*^a^*** | **Comments** |
| --- | --- | --- | --- |
| *cTY R* | cytosolic tyrosine | 97,100 | See Table 2 of main text |
| *cPHE* | cytosolic phenylalanine | 74,300 |  |
| *cTRP* | cytosolic tryptophan | 55,700 |  |
| *pTY R* | tyrosine in the pool of proteins | 1,455,000 |  |
| *pPHE* | phenylalanine in the pool of proteins | 1,112,000 |  |
| *pTRP* | tryptophan in the pool of proteins | 833,000 |  |
| *LDOPA* | l-3,4-dihydroxyphenylalanine | 4.18 | See Table 3 of main text |
| *cDA* | cytosolic dopamine | 41.8 |  |
| *vDA* | vesicular dopamine | 32,500 |  |
| *eDA* | extracellular dopamine | 2 [1–3] |  |
| *cDOPAL* | cytosolic 3,4-dihydroxyphenylacetaldehyde | 2,460 | See Table 3 of main text |
| *cDOPAC* | cytosolic 3,4-dihydroxyphenylacetic acid | 12.3 |  |
| *eDOPAL* | extracellular 3,4-dihydroxyphenylacetaldehyde | 2,000 |  |
| *eDOPAC* | extracellular 3,4-dihydroxyphenylacetic acid | 10 [4,5] |  |
| *HV A* | homovanillic acid | 7 [4,5] |  |
| *5HTP* | 5-hydroxytryptophan | 8.37 | See Table 3 of main text |
| *c5HT* | cytosolic serotonin | 83.7 |  |
| *v5HT* | vesicular serotonin | 65,100 |  |
| *e5HT* | extracellular serotonin | 2 [6–9] |  |
| *c5HIAL* | cytosolic 5-hydroxyindoleacetaldehyde | 4,000 | See Table 3 of main text |
| *c5HIAA* | cytosolic 5-hydroxyindoleacetic acid | 1,970 |  |
| *e5HIAL* | extracellular 5-hydroxyindoleacetaldehyde | 6,400 |  |
| *e5HIAA* | extracellular 5-hydroxyindoleacetic acid | 1,600 [6,8,10,11] |  |
| *KYN* | kynurenine | 58 [10] |  |
| *KYNA* | kynurenic acid | 29 |  |
| *3HK* | 3-hydroxykynurenine | 57 [12] |  |
| *3HAA* | 3-hydroxyanthranilic acid | 19 [13] |  |
| *QUIN* | quinolinic acid | 51 [14] |  |

*^a^*To calculate the actual values in the unit of molar concentration, the conversion factor *f_c_* = 0.7 *ml/g* was used.

**Table B. Independent variables and their concentrations for the baseline level of CORT.** The values are given in relative units, but are consistent with real concentrations found in the literature.

| **Variable** | **Metabolite** | **Concentration*^a^*** |
| --- | --- | --- |
| *sTYR* | serum tyrosine | 100,000 [15] |
| *sPHE* | serum phenylalanine | 72,000 [15] |
| *sTRP* | serum tryptophan | 85,000 [15] |
| *LAT* | l-type amino acid transporter | 1,000*^b^* |
| *TH* | tyrosine hydroxylase | 1,000 |
| *TPH2* | tryptophan hydroxylase 2 | 1,000 |
| *AADC* | aromatic l-amino acid decarboxylase | 1,000 |
| *VMAT2* | vesicular monoamine transporter 2 | 1,000 |
| *MAO* | monoamine oxidase | 1,000 |
| *ALDH* | aldehyde dehydrogenase | 1,000 |
| *COMT* | catechol O-methyltransferase | 1,000 |
| *DAT* | dopamine transporter | 1,000 |
| *SERT* | serotonin transporter | 1,000 |
| *IDO* | indoleamine-2,3-dioxygenase | 1,000 |
| *TDO* | tryptophan-2,3-dioxygenase | 1,000 |
| *KAT* | kynurenine aminotransferase | 1,000 |
| *KYNU* | kynureninase | 1,000 |
| *KMO* | kynurenine-3-monooxygenase | 1,000 |
| *HAAO* | 3-hydroxyanthranilic acid dioxygenase | 1,000 |
| *QPRT* | quinolinate phosphoribosyltransferase | 1,000 |
| *CORT* | corticosterone/cortisol | 1,000 |

*^a^*To calculate the actual values in the unit of molar concentration, the conversion factor *f_c_* = 0.7 *ml/g* was used.

*^b^*Lacking actual values, all proteins were assigned to the same relative number.

**References**

1. Beaufour CC, Le Bihan C, Hamon M, Thiébot M-H. Extracellular dopamine in the rat prefrontal cortex during reward-, punishment-and novelty-associated behaviour. Effects of diazepam. Pharmacol Biochem Behav. 2001;69(1–2):133–42.

2. Jones SR, Gainetdinov RR, Jaber M, Giros B, Wightman RM, Caron MG. Profound neuronal plasticity in response to inactivation of the dopamine transporter. Proc Natl Acad Sci USA. 1998;95(7):4029–34.

3. Westerink BHC, Spaan SJ. On the significance of endogenous 3-methoxytyramine for the effects of centrally acting drugs on dopamine release in the rat brain. J Neurochem. 1982;38(3):680–6.

4. Lavicky J, Dunn AJ. Corticotropin-releasing factor stimulates catecholamine release in hypothalamus and prefrontal cortex in freely moving rats as assessed by microdialysis. J Neurochem. 1993;60(2):602–12.

5. Matsuno K, Matsunaga KH, Mita S. Acute effects of σ ligands on the extracellular DOPAC level in rat frontal cortex and striatum. Neurochem Res. 1995;20(2):233–8.

6. Adell A, Carceller A, Artigas F. Regional distribution of extracellular 5-hydroxytryptamine and 5-hydroxyindoleacetic acid in the brain of freely moving rats. J Neurochem. 1991;56(2):709–12.

7. de Groote L, Olivier B, Westenberg HG. Extracellular serotonin in the prefrontal cortex is limited through terminal 5-HT 1B autoreceptors: a microdialysis study in knockout mice. Psychopharmacology (Berl). 2002;162(4):419–24.

8. Gomez-Merino D, Béquet F, Berthelot M, Chennaoui M, Guezennec CY. Site-dependent effects of an acute intensive exercise on extracellular 5-HT and 5-HIAA levels in rat brain. Neurosci Lett. 2001;301(2):143–6.

9. Mällo T, K\~oiv K, Koppel I, Raudkivi K, Uustare A, Rinken A, et al. Regulation of extracellular serotonin levels and brain-derived neurotrophic factor in rats with high and low exploratory activity. Brain Res. 2008;1194:110–7.

10. Braidy N, Guillemin GJ, Mansour H, Chan-Ling T, Grant R. Changes in kynurenine pathway metabolism in the brain, liver and kidney of aged female Wistar rats. FEBS J. 2011;278(22):4425–34.

11. Kalén P, Strecker RE, Rosengren E, Björklund A. Endogenous release of neuronal serotonin and 5-hydroxyindoleacetic acid in the caudate-putamen of the rat as revealed by intracerebral dialysis coupled to high-performance liquid chromatography with fluorimetric detection. J Neurochem. 1988;51(5):1422–35.

12. Gal EM, Sherman AD. Synthesis and metabolism of L-kynurenine in rat brain. J Neurochem. 1978;30(3):607–13.

13. Baran H, Schwarcz R. Presence of 3-hydroxyanthranilic acid in rat tissues and evidence for its production from anthranilic acid in the brain. J Neurochem. 1990;55(3):738–44.

14. Heyes MP, Markey SP. Quantification of quinolinic acid in rat brain, whole blood, and plasma by gas chromatography and negative chemical ionization mass spectrometry: effects of systemic L-tryptophan administration on brain and blood quinolinic acid concentrations. Anal Biochem. 1988;174(1):349–59.

15. Glaeser BS, Maher TJ, Wurtman RJ. Changes in brain levels of acidic, basic, and neutral amino acids after consumption of single meals containing various proportions of protein. J Neurochem. 1983;41(4):1016–21.
