# Supplementary material for: A model of dopamine and serotonin-kynurenine metabolism in cortisolemia: Implications for depression
Source: PLoS Comput Biol. 2021 May 10;17(5):e1008956. doi: 10.1371/journal.pcbi.1008956 (PMC8136856; doi:10.1371/journal.pcbi.1008956)
Supplement: S4 Supplement — (DOCX) [file pcbi.1008956.s004.docx]

**S4 Supplement. Sensitivity analysis**

The log gains for the baseline model and the model under CORT stress (see Methods) are presented in Tables A and B, respectively. Every independent variable was increased by 10% and the corresponding change in dependent variables at the steady state was measured. The results are similar to those with changes of 1% (not shown). Out of a total of almost 300 gains, almost all are unremarkable. Four somewhat higher values are obtained for CORT, which one might expect, and LAT, which is represented in a very simplified fashion, but even in these cases the gains are only between 30 and 50, which corresponds to 3% and 5% changes if the independent variable is increased by 1%, which is the typical norm.

**Table A. Log gains for metabolites in the dopaminergic pathway with respect to 10% changes in independent variables of the model under baseline levels of CORT.**

| ***X_in_*** | ***cTYR*** | ***cPHE*** | ***LDOPA*** | ***cDA*** | ***vDA*** | ***eDA*** | ***cDOPAL*** | ***cDOPAC*** | ***eDOPAL*** | ***eDOPAC*** | ***HVA*** |
| --- | --- | --- | --- | --- | --- | --- | --- | --- | --- | --- | --- |
| *sTYR* | 18.8 | - | -5.8 | -14.5 | -12.1 | -3.7 | -8.7 | -5.3 | -4.0 | -3.4 | -3.6 |
| *sPHE* | -8.1 | 7.8 | 1.9 | 4.4 | 3.6 | - | 2.1 | 2.1 | - | 1.3 | 1.4 |
| *sTRP* | -7.5 | -5.9 | 3.7 | 10.5 | 8.5 | 2.6 | 6.4 | 2.8 | 3.6 | 1.8 | 1.9 |
| *LAT* | 22.0 | 16.9 | -9.1 | -21.4 | -18.0 | -5.5 | -12.2 | -9.4 | -4.4 | -6.0 | -6.4 |
| *TH* | -1.2 | -1.2 | 6.0 | 16.4 | 13.3 | 3.7 | 9.0 | 5.4 | 3.9 | 3.5 | 3.6 |
| *AADC* | - | - | -6.8 | 6.5 | 5.4 | 1.6 | 3.7 | 2.2 | 1.7 | 1.5 | 1.5 |
| *VMAT2* | - | - | -6.6 | 1.9 | 10.6 | 3.4 | 1.2 | - | 3.4 | 2.5 | 1.1 |
| *MAO* | - | - | 1.1 | -5.2 | -3.4 | -1.4 | 2.8 | 3.0 | 8.4 | 6.6 | 3.3 |
| *ALDH* | - | - | - | -3.7 | -2.3 | - | -5.9 | 4.1 | -6.9 | 3.1 | 2.9 |
| *COMT* | - | - | - | -3.4 | -2.7 | - | -2.9 | -7.1 | -2.5 | -8.1 | 1.4 |
| *DAT* | - | - | - | 7.8 | 3.9 | -3.7 | 4.3 | 2.4 | -3.6 | -2.3 | - |
| *SERT* | - | - | - | -2.0 | -1.5 | -1.7 | -1.2 | - | -1.7 | -1.3 | - |
| *KMO* | - | - | - | - | - | - | 1.3 | - | 1.6 | - | - |
| *HAAO* | - | - | - | - | - | - | - | - | -1.2 | - | - |
| *CORT* | 23.3 | 17.5 | -3.3 | -8.9 | -8.4 | -2.4 | 7.0 | -3.4 | 19.1 | 5.8 | -7.3 |

Only changes whose absolute values are equal or greater than 1% are displayed.

Considering the gains in Table A, the first interesting result is the impact of an increase in *sTYR* on the entire system. Although an augmentation of 10% in the serum tyrosine concentration is expected to induce a corresponding increase in the dopamine biosynthesis, decreases are observed in *cDA* (↓14.5%) and *vDA* (↓12.1%). These drops are in agreement with the fact that, under physiological conditions, TH is already saturated and inhibited by tyrosine at its physiological concentration [1]. Although the magnitudes of the log gains are different, a similar response was found for a small increase in LAT. This increase means that the more amino acid transporters are available, the more cytosolic amino acids are present and, consequently, a similar effect is seen for the serum concentration of amino acids.

**Table B. Log gains for metabolites in the serotonergic-kynurenine pathway with respect to changes in independent variables of the model under baseline levels of CORT.**

| ***X_in_*** | ***cTRP*** | ***5HTP*** | ***c5HT*** | ***v5HT*** | ***e5HT*** | ***c5HIAL*** | ***c5HIAA*** | ***e5HIAL*** | ***e5HIAA*** | ***KYN*** | ***KYNA*** | ***3HK*** | ***3HAA*** | ***QUIN*** |
| --- | --- | --- | --- | --- | --- | --- | --- | --- | --- | --- | --- | --- | --- | --- |
| *sPHE* | -17.4 | -2.7 | -1.9 | -1.6 | - | -4.3 | -3.4 | -1.8 | -1.3 | -3.9 | -3.0 | -3.5 | -2.6 | -2.0 |
| *sTRP* | 28.5 | 3.7 | 2.5 | 2.2 | - | 5.9 | 4.6 | 2.4 | 1.7 | 5.4 | 4.1 | 4.7 | 3.5 | 2.7 |
| *LAT* | 49.1 | 5.9 | 4.0 | 3.5 | 1.4 | 9.5 | 7.5 | 3.8 | 2.7 | 8.7 | 6.6 | 7.6 | 5.6 | 4.3 |
| *TPH* | -11.7 | 9.4 | 6.2 | 5.4 | 2.1 | 10.4 | 12.2 | 2.6 | 4.7 | -2.6 | -2.0 | -2.3 | -1.7 | -1.3 |
| *AADC* | - | -7.1 | 1.5 | 1.3 | - | 2.6 | 2.8 | - | 1.1 | - | - | - | - | - |
| *VMAT2* | 1.1 | -3.1 | - | 7.2 | 2.8 | -1.0 | -1.2 | 2.6 | - | - | - | - | - | - |
| *MAO* | -2.9 | - | -4.7 | -1.7 | -1.0 | 9.5 | 10.5 | 9.5 | 5.4 | - | - | - | - | - |
| *ALDH* | - | - | - | - | - | -10.0 | - | -7.7 | - | - | - | - | - | - |
| *DAT* | - | - | - | - | - | -1.6 | -1.7 | - | - | - | - | - | - | - |
| *SERT* | -1.8 | 1.2 | 2.9 | - | -6.2 | 5.0 | 5.6 | -5.7 | - | - | - | - | - | - |
| *IDO* | -12.8 | -4.4 | -3.0 | -2.6 | -1.0 | -4.0 | -5.7 | - | -2.4 | 4.8 | 3.7 | 4.2 | 3.1 | 2.4 |
| *TDO* | -12.8 | -4.4 | -3.0 | -2.6 | -1.0 | -4.0 | -5.7 | - | -2.4 | 4.8 | 3.7 | 4.2 | 3.1 | 2.4 |
| *KAT* | - | - | - | - | - | - | - | - | - | - | 7.0 | - | - | - |
| *KYNU* | - | - | - | - | - | 1.4 | - | 1.0 | - | - | - | -2.5 | 7.2 | 5.6 |
| *KMO* | - | - | - | - | - | 2.4 | - | 1.8 | - | - | - | 8.5 | 6.2 | 4.8 |
| *HAAO* | - | - | - | - | - | -1.7 | - | -1.3 | - | - | - | - | -6.8 | 2.0 |
| *QPRT* | - | - | - | - | - | - | - | - | - | - | - | - | -2.1 | -8.7 |
| *CORT* | 40.5 | - | -3.3 | -2.3 | -1.0 | 27.6 | - | 25.0 | 4.6 | 12.9 | 9.8 | 16 | 20.1 | 23.2 |

Only changes whose absolute values are equal or greater than 1% are shown.

The results in both Tables A and B demonstrate the effects of a small increase in the concentration of serum PHE (*sPHE*) with regard to the biosynthesis of DA and 5-HT. PHE also inhibits TH activity, but this effect was neglected in this model since the physiological concentration of *cPHE* is relatively low in comparison to the observed concentration that causes inhibition of TH [1]. As TH is already saturated and inhibited by TYR, the small increase of *sPHE* is followed by an increase in *cPHE* (↑7.8%), which leads to competition with *cTYR* for TH. This decrease in cytosolic TYR (↓8.1%) produces an immediate increase in DA synthesis, since the substrate inhibition of TH is reduced. This is true for small changes around the operation point but it is not what happens in phenylketonuria (PKU), a genetic disorder in which a mutation in the phenylalanine hydroxylase gene causes an accumulation of serum phenylalanine of about 10 times the normal range [2]. Even a modest impairment in this enzyme causes an accumulation of serum phenylalanine and a consequent decrease in tyrosine and tryptophan uptake, due to the high affinity of phenylalanine for the amino acid transporters in the BBB [3].

Notice, however, that the impact of the small increase in serum phenylalanine immediately triggers a change in *cTRP* (↓17.4%) and, consequently, in the cytosolic level of serotonin. Although TPH2 also exhibits substrate inhibition for high levels of tryptophan, this enzyme is not saturated under physiological conditions, in contrast to TH. By contrast, we find an increase in *c5HT* (↑2.5%) and *v5HT* (↑2.2%) due to an increase of 10% in blood tryptophan. This behavior is in agreement with similar models in the literature [4], although we have not found the same sensitivities for this relationship.

Another protein performing a key role in both systems is ALDH. This enzyme is the major catalyst for converting the neurotoxic aldehydes produced by the catabolism of DA and 5-HT into less toxic metabolites, such as DOPAC and 5-HIAA. For the dopaminergic terminal, the log gain analysis indicated an increase in *cDOPAC* (↑4.1%), resulting from the reduction in *cDOPAL* (↓5.9%). A similar result was found in the serotonergic terminal model, which responded with decreases in *c5HIAL* (↓7.7%).

According to the model, the enzymes involved in the metabolism of kynurenine do not seem to have a significant impact either on the dopaminergic or the serotonergic metabolism when the systems is operating at the baseline levels of CORT, except for IDO‑TDO in Table B. However, it is worth highlighting that IDO-TDO presents a deleterious effect on the availability of cytosolic tryptophan, similar to the one found in the excesses of phenylalanine. In absolute terms, the log gain for *cTRP* with respect to IDO-TDO (↓12.8%) is slightly smaller than *sPHE* (↓17.4%). However, this may be somewhat misleading, since this model is semi-quantitative, due to numerous assumptions; thus, additional lab experiments will be needed to validate or refine this result. The enzyme KMO also stands out in the log gain analyses. A small increase in *KMO* alone is responsible for the increase in *3HK* (↑8.5%), *3HAA* (↑6.2%), and *QUIN* (↑4.8%). This result makes KMO a potential target for a pharmacological intervention.

Finally, regarding the role of CORT, we see in Table A a deleterious effect in *LDOPA* (↓3.3%) and consequent decreases in *cDA* (↓8.9%) and *vDA* (↓8.4%). In addition, CORT leads to increases in the aldehydes *cDOPAL* (↑7%) and *eDOPAL* (↑19.1%). Table B furthermore indicates the impact of *CORT* on the metabolism of serotonin, by decreasing *c5HT* (↓3.3%), and *v5HT* (↓2.3%), which is to be expected since CORT was modeled to inhibit TPH2 [5]. Also, the increment in CORT significantly increases the levels of the neurotoxic KYN metabolites *3HK* (↑16%), *3HAA* (↑20.1%), and *QUIN* (↑23.2%). This was also likely to be observed because, according to our model, *CORT* raises the activity of *IDO*, *KAT*, and *HAAO* [6,7]. These enzymes are involved in the production of KYN and its metabolites, and some of them are known to impair ALDH activity [8]; ultimately, they lead to increases in DOPAL and 5-HIAL.

Taken together, the results observed in the log gain analysis are not only mathematically acceptable but also physiologically consistent with experimental findings on dopaminergic, serotonergic, and kynurenine metabolism, especially under the influence of CORT.

Tables C and D show the most relevant results of the sensitivity analysis for metabolites in both terminals due to 10% changes in the kinetic orders. Notice that the vast majority of the gains are below the 5-fold range, indicating the robustness of our model.

**Table C. Sensitivity analysis for metabolites in the dopaminergic pathway with respect to 10% changes in the kinetic orders.**

| ***Kinetic***  ***Order*** | ***cTYR*** | ***cPHE*** | ***LDOPA*** | ***cDA*** | ***vDA*** | ***eDA*** | ***cDOPAL*** | ***cDOPAC*** | ***eDOPAL*** | ***eDOPAC*** | ***HVA*** |
| --- | --- | --- | --- | --- | --- | --- | --- | --- | --- | --- | --- |
| $f_{1,1}$ | 579.5 | - | -49.9 | -84.2 | -78 | -36.1 | -66.6 | -52.4 | -37.5 | -34.8 | -38 |
| $f_{1,2}$ | 160.5 | - | -29.1 | -59.8 | -52.7 | -19.8 | -41.3 | -29.3 | -20.6 | -18.9 | -20.4 |
| $f_{1,3}$ | 133.3 | - | -26.3 | -55.3 | -48.4 | -17.7 | -37.5 | -26.2 | -18.4 | -16.8 | -18.2 |
| $f_{1,4}$ | 271.9 | - | -37.7 | -71.5 | -64.3 | -26.3 | -52.3 | -38.7 | -27.3 | -25.1 | -27.4 |
| $f_{2,1}$ | -11 | - | 50.8 | 182 | 136 | 28.6 | 77.5 | 38.6 | 29.6 | 25.6 | 25.3 |
| $f_{2,2}$ | - | - | 22.6 | 68.9 | 54.2 | 13.5 | 34.3 | 19.2 | 14.1 | 12.4 | 12.7 |
| $f_{2,6}$ | -10.3 | - | 47.3 | 166.6 | 125.2 | 26.9 | 72.2 | 36.4 | 27.8 | 24.1 | 23.9 |
| $f_{3,1}$ | - | 20.4 | - | - | - | - | - | - | - | - | - |
| $f_{3,2}$ | - | 155 | -14.5 | -33.8 | -28.7 | - | -21.2 | -14 | - | - | - |
| $f_{3,3}$ | - | 93 | -10.5 | -25.3 | -21.3 | - | -15.5 | -10 | - | - | - |
| $f_{3,4}$ | - | 182 | -15.9 | -36.5 | -31.2 | -10.4 | -23.2 | -15.4 | -10.8 | - | -10.5 |
| $f_{4,1}$ | - | -14.1 | - | 18.8 | 15.3 | - | 10.3 | - | - | - | - |
| $f_{4,5}$ | - | -14.8 | - | 19.9 | 16.2 | - | 10.9 | - | - | - | - |
| $f_{7,2}$ | - | - | - | -20.9 | -17.5 | - | -12.6 | - | - | - | - |
| $f_{9,1}$ | - | - | - | -10.1 | 18.9 | - | - | - | - | - | - |
| $f_{9,2}$ | - | - | - | -22.1 | 47.9 | 12.2 | -13.3 | - | 11.8 | - | - |
| $f_{9,3}$ | - | - | - | - | - | - | - | - | - | - | - |
| $f_{10,1}$ | - | - | - | -12.2 | - | - | 15.6 | 12.6 | - | - | - |
| $f_{10,2}$ | - | - | - | -25 | -16.3 | - | 35.7 | 28.5 | - | - | 14 |
| $f_{10,3}$ | - | - | - | -22.7 | -14.8 | - | 31.9 | 25.5 | - | - | 12.6 |
| $f_{11,1}$ | - | - | - | -20.7 | -13.4 | - | -33.7 | 30.1 | - | - | 15.1 |
| $f_{11,6}$ | - | - | - | -22.1 | -14.4 | - | -35.7 | 32.4 | - | - | 16.2 |
| $f_{13,1}$ | - | - | - | - | - | - | - | - | -36.9 | 20.4 | - |
| $f_{13,6}$ | - | - | - | - | - | - | - | - | -39.3 | 22.1 | - |
| $f_{15,3}$ | - | - | - | - | - | - | - | -15.9 | - | - | - |
| $f_{17,1}$ | - | - | - | -20.4 | -46 | 11.1 | -12.2 | - | 10.8 | - | - |
| $f_{21,2}$ | - | - | - | - | - | - | - | - | 23.9 | 16.9 | - |
| $f_{21,3}$ | - | - | - | -16.9 | -11.3 | - | - | - | 86.5 | 58 | 13.6 |
| $f_{22,1}$ | - | - | - | - | - | - | - | -14.9 | - | - | - |
| $f_{22,3}$ | - | - | - | - | - | - | - | -39.3 | - | - | 11 |
| $f_{23,1}$ | - | - | - | - | - | - | - | - | - | -16.7 | - |
| $f_{23,3}$ | - | - | - | - | - | - | - | - | -13.1 | -45.3 | - |
| $f_{24,1}$ | - | - | - | - | - | - | - | - | - | - | -15.3 |
| $f_{25,1}$ | -68 | - | 51.3 | 184.7 | 137.9 | 28.9 | 78.4 | 38.9 | 29.9 | 25.9 | 25.5 |
| $f_{26,1}$ | - | -62.7 | 19.3 | 57.5 | 45.5 | 11.6 | 29.2 | 16.6 | 12.1 | 10.7 | 11 |
| $f_{27,1}$ | 64.4 | - | -16.4 | -37.5 | -32.1 | -10.8 | -23.8 | -15.9 | -11.2 | -10.1 | -10.9 |
| $f_{27,2}$ | 18.9 | - | - | -15.1 | -12.6 | - | - | - | - | - | - |
| $f_{28,1}$ | - | 42.9 | - | -14.8 | -12.3 | - | - | - | - | - | - |
| $f_{28,2}$ | - | 13.7 | - | - | - | - | - | - | - | - | - |
| $f_{29,1}$ | -12.9 | - | - | 13.8 | 11.3 | - | - | - | - | - | - |
| $f_{53,3}$ | - | - | - | - | - | - | 10.9 | - | 13.1 | - | - |

Only changes whose absolute values are equal or greater than 10% are shown.

**Table D. Sensitivity analysis for metabolites in the serotonergic-kynurenine pathway with respect to 10% changes in the kinetic orders.**

| ***Kinetic***  ***Order*** | ***cTRP*** | ***5HTP*** | ***c5HT*** | ***v5HT*** | ***e5HT*** | ***c5HIAL*** | ***c5HIAA*** | ***e5HIAL*** | ***e5HIAA*** | ***KYN*** | ***KYNA*** | ***3HK*** | ***3HAA*** | ***QUIN*** |
| --- | --- | --- | --- | --- | --- | --- | --- | --- | --- | --- | --- | --- | --- | --- |
| $f_{32,1}$ | 65.6 | - | - | - | - | 12.2 | - | - | - | 11.1 | - | - | - | - |
| $f_{32,2}$ | 446.4 | 27.5 | 17.9 | 15.5 | - | 46.5 | 35.2 | 17.2 | 12.2 | 42.5 | 31.3 | 36.5 | 26.1 | 19.7 |
| $f_{32,3}$ | 800 | 37 | 23.6 | 20.5 | - | 63.5 | 47.4 | 22.8 | 16 | 58.2 | 42.2 | 49.4 | 34.8 | 26.1 |
| $f_{32,4}$ | 782.9 | 36.6 | 23.4 | 20.3 | - | 62.9 | 46.9 | 22.6 | 15.9 | 57.5 | 41.8 | 48.9 | 34.5 | 25.8 |
| $f_{33,1}$ | -24.5 | 20.6 | 13.3 | 11.7 | - | 22.6 | 27.1 | - | 10.2 | - | - | - | - | - |
| $f_{33,2}$ | -16.6 | 13.5 | - | - | - | 14.8 | 17.6 | - | - | - | - | - | - | - |
| $f_{33,4}$ | -14.8 | 12 | - | - | - | 13.2 | 15.6 | - | - | - | - | - | - | - |
| $f_{33,5}$ | -32.1 | 27.9 | 17.7 | 15.6 | - | 30.4 | 36.8 | - | 13.6 | - | - | - | - | - |
| $f_{34,1}$ | - | -12.8 | - | - | - | - | - | - | - | - | - | - | - | - |
| $f_{34,2}$ | - | -51.1 | 10.1 | 12.7 | - | 18.9 | 20.1 | - | - | - | - | - | - | - |
| $f_{37,1}$ | - | - | -11.8 | - | - | -20.3 | -21.4 | - | - | - | - | - | - | - |
| $f_{37,2}$ | - | - | - | - | - | -16.3 | -17.2 | - | - | - | - | - | - | - |
| $f_{38,1}$ | -10.6 | - | -17.2 | - | - | 55.7 | 62.8 | - | 19.7 | - | - | - | - | - |
| $f_{38,2}$ | - | - | - | - | - | - | - | - | - | - | - | - | - | - |
| $f_{38,3}$ | -18 | - | -27.8 | -10.3 | - | 100 | 114.8 | - | 33.2 | - | - | - | - | - |
| $f_{39,1}$ | - | - | - | - | - | -50.2 | - | - | - | - | - | - | - | - |
| $f_{39,2}$ | - | - | - | - | - | -21.3 | - | - | - | - | - | - | - | - |
| $f_{39,6}$ | - | - | - | - | - | -52 | - | - | - | - | - | - | - | - |
| $f_{40,1}$ | - | - | - | - | - | -19.6 | -48.7 | - | - | - | - | - | - | - |
| $f_{40,2}$ | - | - | - | - | - | -21.2 | -51.8 | - | - | - | - | - | - | - |
| $f_{41,1}$ | - | 10.9 | - | 19.2 | - | -10.3 | -11.2 | - | - | - | - | - | - | - |
| $f_{41,2}$ | - | 28.2 | -15.1 | 54.6 | 18.7 | -25.1 | -26.9 | 15.2 | - | - | - | - | - | - |
| $f_{43,1}$ | - | -32.1 | - | -40.8 | 23.3 | - | - | 22.7 | - | - | - | - | - | - |
| $f_{47,2}$ | - | - | - | - | - | - | - | 27.8 | - | - | - | - | - | - |
| $f_{47,3}$ | - | - | - | - | - | -12.6 | -13.2 | 86.5 | 10.4 | - | - | - | - | - |
| $f_{48,1}$ | - | - | - | - | - | - | - | -43.1 | - | - | - | - | - | - |
| $f_{48,2}$ | - | - | - | - | - | - | - | -17.1 | - | - | - | - | - | - |
| $f_{48,6}$ | - | - | - | - | - | - | - | -44.7 | - | - | - | - | - | - |
| $f_{51,1}$ | - | - | - | - | - | - | - | -15.7 | -48.3 | - | - | - | - | - |
| $f_{52,1}$ | -35.1 | -12.8 | - | - | - | -12 | -16.4 | - | - | 13.7 | 10.4 | 12 | - | - |
| $f_{52,2}$ | -16.4 | - | - | - | - | - | - | - | - | - | - | - | - | - |
| $f_{52,3}$ | -56.4 | -22.1 | -15.5 | -13.6 | - | -21.5 | -28 | - | -12.4 | 23.3 | 17.4 | 20.2 | 14.7 | 11.2 |
| $f_{53,1}$ | - | - | - | - | - | 10.5 | - | - | - | - | - | 40.6 | 28.8 | 21.7 |
| $f_{53,2}$ | - | - | - | - | - | - | - | - | - | - | - | 11.2 | - | - |
| $f_{53,3}$ | - | - | - | - | - | 20.6 | - | 15 | - | - | - | 45.6 | 83.7 | 60.2 |
| $f_{54,3}$ | - | - | - | - | - | - | - | - | - | - | - | -19.5 | 61.8 | 45.2 |
| $f_{55,1}$ | - | - | - | - | - | - | - | - | - | - | - | - | -18.3 | - |
| $f_{55,3}$ | - | - | - | - | - | - | - | - | - | - | - | - | -18.5 | - |
| $f_{56,1}$ | - | - | - | - | - | - | - | - | - | - | 32.6 | - | - | - |
| $f_{57,1}$ | -24.4 | - | - | - | - | - | - | - | - | -27 | -21.5 | -24.4 | -18.8 | -14.9 |
| $f_{58,1}$ | - | - | - | - | - | - | - | - | - | - | - | -19.3 | -14.8 | -11.7 |
| $f_{59,1}$ | - | - | - | - | - | - | - | - | - | - | -21.8 | - | - | - |
| $f_{61,1}$ | - | - | - | - | - | - | - | - | - | - | - | - | - | -28.6 |
| $f_{61,2}$ | - | - | - | - | - | - | - | - | - | - | - | - | - | -28 |
| $f_{62,1}$ | -39.2 | - | - | - | - | -10.8 | - | - | - | - | - | - | - | - |
| $f_{63,1}$ | 11 | - | - | - | - | - | - | - | - | - | - | - | - | - |

Only changes whose absolute values are equal or greater than 1% are shown.

Abbreviations: *3HAA*, 3-hydroxyanthranilic acid; *3HK*, 3-hydroxyanthranilic acid; *5HTP*, 5-hydroxytryptophan; *AADC*, aromatic l-amino acid decarboxylase; *ALDH*, aldehyde dehydrogenase; *c5HT*, cytosolic serotonin; *c5HIAA*, cytosolic 5-hydroxyindoleacetic acid; *c5HIAL*, cytosolic 5-hydroxyindoleacetaldehyde; *cDA*, cytosolic dopamine; *cDOPAC*, cytosolic 3,4-dihydroxyphenylacetic acid; *cDOPAL*, cytosolic 3,4-dihydroxyphenylacetaldehyde; *COMT*, catechol O-methyltransferase; *CORT*, corticosterone/cortisol; *cPHE*, cytosolic phenylalanine; *cTRP*, cytosolic tryptophan; *cTYR*, cytosolic tyrosine; *DAT*, dopamine transporter; *e5HT*, extracellular serotonin; *e5HIAL*, extracellular 5-hydroxyindoleacetaldehyde; *e5HIAA*, extracellular 5-hydroxyindoleacetic acid; *eDA*, extracellular dopamine; *eDOPAC*, extracellular 3,4-dihydroxyphenylacetic acid; *eDOPAL*, extracellular 3,4-dihydroxyphenylacetaldehyde; *HAAO*, 3-hydroxyanthranilic acid dioxygenase; *HVA*, homovanillic acid; *IDO*, indoleamine-2,3-dioxygenase; *KAT*, kynurenine aminotransferase; *KMO*, kynurenine-3-monooxygenase; *KYN*, kynurenine; *KYNA*, kynurenic acid; *KYNU*, kynureninase; *LAT*, l-type amino acid transporter; *LDOPA*, l-3,4-dihydroxyphenylalanine; *MAO*, monoamine oxidase; *QPRT*, quinolinate phosphoribosyltransferase; *QUIN*, quinolinic acid; *SERT*, serotonin transporter; *sPHE*, serum phenylalanine; *sTRP*, serum tryptophan; *sTYR*, serum tyrosine; *TDO*, tryptophan-2,3-dioxygenase; *TH*, tyrosine hydroxylase; *TPH2*, tryptophan hydroxylase 2; *v5HT*, vesicular serotonin; *vDA*, vesicular dopamine; *VMAT2*, vesicular monoamine transporter 2.

**References**

1. Ribeiro P, Pigeon D, Kaufman S. The hydroxylation of phenylalanine and tyrosine by tyrosine hydroxylase from cultured pheochromocytoma cells. J Biol Chem. 1991;266(24):16207–11.

2. Blau N, van Spronsen FJ, Levy HL. Phenylketonuria. Lancet. 2010;376(9750):1417–27.

3. Smith QR, Momma S, Aoyagi M, Rapoport SI. Kinetics of neutral amino acid transport across the blood-brain barrier. J Neurochem. 1987;49(5):1651–8.

4. Best J, Duncan W, Sadre-Marandi F, Hashemi P, Nijhout HF, Reed M. Autoreceptor control of serotonin dynamics. BMC Neurosci. 2020;

5. Chen Y, Xu H, Zhu M, Liu K, Lin B, Luo R, et al. Stress inhibits tryptophan hydroxylase expression in a rat model of depression. Oncotarget. 2017;8(38):63247.

6. Couch Y, Anthony DC, Dolgov O, Revischin A, Festoff B, Santos AI, et al. Microglial activation, increased TNF and SERT expression in the prefrontal cortex define stress-altered behaviour in mice susceptible to anhedonia. Brain Behav Immun. 2013;29:136–46.

7. Martín-Hernández D, Tendilla-Beltrán H, Madrigal JLM, García-Bueno B, Leza JC, Caso JR. Chronic mild stress alters kynurenine pathways changing the glutamate neurotransmission in frontal cortex of Rats. Mol Neurobiol. 2019;56(1):490–501.

8. Badawy AA-B, Morgan CJ. Tryptophan metabolites as potent inhibitors of aldehyde dehydrogenase activity and potential alcoholism-aversion therapeutic agents. In: International Congress Series. 2007. p. 344–51.
